# Personalized Models of Biological Barriers and Their Diseases: Recent Progress with Organs‐On‐Chips

**DOI:** 10.1002/adbi.202500536

**Published:** 2026-02-11

**Authors:** Franziska Buck, Jeroen Bugter, Gizem Yorukoglu, Mina Kazemzadeh Dastjerd, Thomas E. Winkler

**Affiliations:** ^1^ Institute of Microtechnology (IMT) & Center of Pharmaceutical Engineering (PVZ) Technische Universität Braunschweig Braunschweig Germany; ^2^ Department of Micro‐ and Nanosystems (MST) SciLifeLab KTH Royal Institute of Technology Stockholm Sweden; ^3^ Department of Micro‐ and Nanosystems (MST), Digital Futures & SciLifeLab KTH Royal Institute of Technology Stockholm Sweden; ^4^ Institute of Microtechnology (IMT) Technische Universität Braunschweig Braunschweig Germany

**Keywords:** human‐induced pluripotent stem cells, microphysiological systems, organ‐on‐a‐chip, personalized medicine, tissue barriers

## Abstract

Barrier tissues—epithelial and endothelial interfaces that compartmentalize the human body—govern molecular exchange, immune surveillance, and organ homeostasis. Their dysfunction is central to disorders ranging from dermatitis to neurodegeneration. Conventional static cultures fail to capture the relevant microenvironment and typically rely on cell lines that overlook patient‐specific genetics. Organs‐on‐chips (OoCs), by contrast, can recapitulate barrier‐specific flow, biomechanics, chemical gradients, and a multicellular architecture. Additionally, incorporating primary or induced pluripotent stem cell (iPSC)‐derived cells into OoCs can open new avenues for precision medicine. This review surveys the architectural diversity and physiological functions of human barrier systems and explores how OoC platforms—especially those using patient‐derived cells—are advancing barrier disease modeling. It reveals similar core features but also unique barrier characteristics requiring specific adaptations, resulting in varied progress across systems, and continued refinement of iPSC differentiation protocols and OoC engineering is needed overall. Nevertheless, existing biological and technological advances already offer substantial, untapped opportunities to create physiologically relevant, patient‐specific disease models and drug‐testing platforms, bridging the gap between fundamental biology and translational medicine.

## Graphical abstract

Buck and Bugter et al. explore the architectural diversity and physiological functions of human barrier systems and reveal how organ‐on‐chip platforms, particularly those integrating patient‐derived cells, are advancing barrier disease modeling. They highlight how emerging biological and technological advances can be used to bridge the gap between fundamental biology and translational medicine. Created with BioRender.com.

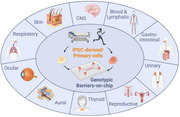

**Abbreviations**
ALS: amyotrophic lateral sclerosis
AT1 and AT2: alveolar type 1 and 2 cells
BBB: blood–brain barrier
BLB: blood–labyrinth barrier
BRB: blood‐retinal barrier
CD: Crohn's disease
CF: cystic fibrosis
CFTR: cystic fibrosis transmembrane conductance regulator
CPAP: continuous positive airway pressure
CSF: cerebrospinal fluid
ECM: extracellular matrix
EC: endothelial cell
GI: gastrointestinal
HGPS: Hutchinson‐Gilford progeria syndrome
HUVEC: human umbilical vein endothelial cell
IBD: inflammatory bowel disease
IL: interleukin
iPSC: induced pluripotent stem cell
iPSC‐*[cell]*
: iPSC‐derived *[cell]*‐like cell
LPS: lipopolysaccharide
MSC: mesenchymal stromal cell
OoC: organ‐on‐chip
PC: pericyte
PDMS: polydimethylsiloxane
RPE: retinal pigment epithelium
SMC: smooth muscle cells
TEER: transepithelial/‐endothelial electrical resistance
UC: ulcerative colitis
VEGF: vascular endothelial growth factor

## 1 Introduction

Tissue barriers shield internal areas of our human bodies from the outside or separate different internal compartments from each other. Vital for organ function, the barriers are formed by epithelial and endothelial cell layers that can regulate passage of ions and solutes, including nutrients or waste products. On a molecular level, the variety and differing abundance of cell–cell junctions, predominately tight junctions, determine the paracellular transport characteristics of a barrier tissue, with selective transmembrane proteins regulating transcellular transport [1, 2]. The asymmetric distribution of these junction and transporter proteins is one aspect of cell polarity, which creates characteristic membrane domains of barrier cells oriented either toward an internal tissue or facing an external or luminal space. These molecular mechanisms give rise to a wide variety of functionality. Our most exposed epithelial barrier, the skin, also serves to control fluid loss [3, 4]. For barriers partially exposed to the outside environment, epithelial layers generate mucous membranes to further shield the underlying tissue and to maintain immensely diverse microbiomes [5, 6, 7]. With its local adaptations, the epithelium's responsibilities range from removing airborne microorganisms and particles in the airway to facilitating digestion in the intestine [4]. Endothelium, meanwhile, serves as the interior surface of our circulatory system vessels [8]. Some examples, such as the blood–brain barrier (BBB), are especially selective so as to strongly limit the entry of all potentially toxic agents and support immune privilege of the relevant organs [2, 9]. Others are conversely much leakier, like lymphatic vessels that help filter excess blood plasma and antigens from tissue [10]. As critical as barrier function is to homeostasis, impaired barrier functions are associated with a variety of disorders, including neurological, gastrointestinal (GI), metabolic, as well as infectious diseases [2, 9]. It is thus imperative to understand and—given analytical and ethical limitations in vivo—to model, in vitro, human tissue barriers. Such models require two key components going forward: modeling of not only the cells but also their unique microenvironment, and modeling of not only general mammalian biology but also human‐ and even patient‐specific response.

In this review, we will first establish a basic overview of, and definitions for, these two key components and their modeling, that is, the microenvironment using microfluidic engineering and patient‐specific response using patient‐derived cell models. We then cover personalized in vitro models for the different epithelial and endothelial barriers across the human body using these technologies. We organize the review by barrier system: the circulatory blood and lymphatic vessels that interconnect and form part of all other organs, followed by the specialized barriers of the central nervous system, skin, ocular and aural systems, respiratory tract, GI and urinary systems, reproductive organs, and the thyroid. While aiming for a comprehensive overview, we nonetheless emphasize barriers with more active in vitro model research (while excluding barriers with less disease relevance like serous membranes, as well as those where barrier function is not defined by endothelial or epithelial cells, such as the synovial membrane). Across these barrier systems, we briefly discuss their basic function and structure, the current state of relevant personalized cell sources, and the potential benefits of dynamic in vitro microenvironments. We then review notable model systems from the literature, focusing on personalized models for disorders with high germline genetic correlation where they may be especially impactful, while generally excluding those primarily driven by environmental agents and/or somatic mutations (e.g., infection or cancer, respectively). Subsequently, we consider multi‐organ chips that connect and integrate such barrier models. Finally, we provide a perspective on technological gaps and advances that are important for functionality and physiological relevance across these models and conclude with a section on efforts and challenges in standardization and translation.

## 2 Background

**FIGURE 1:**
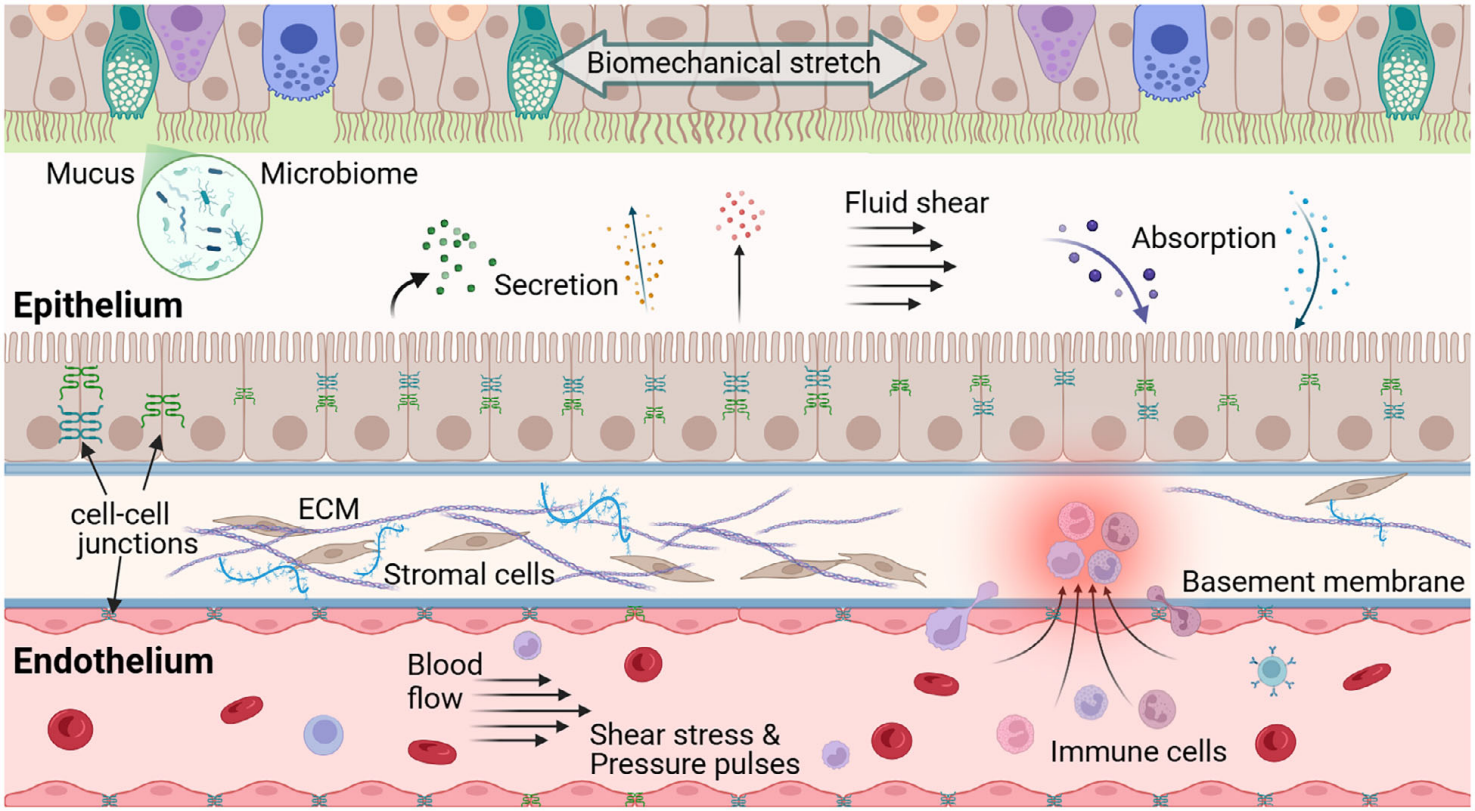
Key biophysical, biochemical, and biological complexity of epithelial and endothelial barriers. Epithelial barriers in particular are characterized by morphological (not shown) and cellular heterogeneity. All barrier tissues are subject to a wide range of secretion and absorption processes, and interact closely with surrounding immune cells and/or microbiota, as well as with their underlying basement membrane and the extracellular matrix (ECM)‐embedded stromal cells underneath. Alongside mechanical forces in the form of fluid flow and stretch (e.g., blood pressure or peristalsis), this highly dynamic environment shapes barrier integrity and tissue responses—and should thus be recapitulated in vitro. Created with BioRender.com.

Barrier functions, like most organ functions, are influenced and modulated by frequently interdependent biochemical and biophysical cues such as tissue geometry, mechanical stimulation like stretch, interaction of different cell types, stiffness and chemical make‐up of the extracellular matrix (ECM), and more (Figure 1) [2, 9]. Barriers are, moreover, unique in that they are often subject to flow shear in vivo. Early models of biological barriers were established on permeable plastic membranes, supporting the formation of polarized cellular monolayers. Still popular today (e.g., Transwell), this facilitates compartmentalized analysis, a basic requirement to study key barrier properties like uptake, secretion, transport, and filtration—all of which may be directional. It also facilitates measurement of the inherent barrier integrity by applying molecular tracer diffusion analysis (high sensitivity, especially in leakier monolayers) or electrical measurements of ionic permeability (transepithelial/‐endothelial electrical resistance; TEER; rapid and real‐time) [2]. Simple cell layers on permeable supports, however, lack many, if not most, aspects of the aforementioned tissue microenvironment.

Advances in microfabrication technologies and biomaterials have enabled compartmentalization in novel microfluidic devices—organs‐on‐chips (OoC)—that approximate organ‐level physiology in an engineered physico‐chemical microenvironment [11]. Accessibility of all compartments in these devices facilitates permeability analysis akin to Transwells, while design flexibility allows tuning several aspects with importance for fundamental tissue functions, including fluidic flow, gradients, actuation, and mechanical cues [2, 11]. Typical OoCs are constructed from transparent engineering polymers and labware‐type plastics; the elastomeric polydimethylsiloxane (PDMS) remains a notably versatile and popular, if sometimes controversial, choice [12]. Biocompatible and physiologically relevant natural hydrogels or synthetic hydrogels with tunable mechanical properties can conversely provide bio‐scaffolds resembling ECM inside these microfluidic housings [2, 11]. The more in‐depth fabrication aspects are outside the scope of our review, and we refer the interested reader to other reviews cited here [13].

Considering barrier tissues, three main OoC strategies are available [2]. The first is to rely on biological self‐organization in a micro‐3D culture, with the ratio of matrix and cells defining the range from vascularized or epithelialized ECM to organoids. Such approaches are, however, difficult to control regarding reproducibility in, for example, barrier network organization, and their lack of defined compartmentalization poses analytical challenges in terms of cross‐barrier transport [11]. To allow regulated analyses of communication between and across different cell types in OoCs, an engineered boundary or interface is thus preferred to facilitate well‐defined organization and separation of different cell types (though a certain level of structural simplification, and thus reduction in biological fidelity, is unavoidable here). One common option is to establish a hydrogel/fluid interface to culture cells on, with the hydrogel serving either as an overly thick and acellular basement membrane or as a cellularized bulk tissue. This approach is limiting with regard to compound diffusion times or tissue compartment access, respectively. In practical terms, two or more channels/compartments are often arranged side‐by‐side here, with the hydrogel confined by micropillars [14] or similar capillary barriers [15], which we will thus term “lateral/hydrogel.” Alternatively, interfaces can be established to more closely mimic our bodies' 3D vessels—by, for example, casting hydrogel around a needle template—in an approach we term “tubular hydrogel.” The final common OoC strategy relies on compartmentalization using a thin (micron‐scale) and permeable membrane to support barrier cell culture, separating two microfluidic channels (above and below)—a design we term “vertical/membrane.” The membrane is most commonly track‐etched plastic akin to Transwells (i.e., stiffer, less permeable, and still thicker than basement membrane), but can be alternatively micro‐engineered from, for example, flexible PDMS, or even assembled from ECM biomaterials [2, 16]. These designs will form the basis for a majority of the OoC examples encountered throughout.

Biological model function is nevertheless determined by the cells inside them. Toward personalized models, this naturally requires cells from patients. The direct use of primary patient materials from the tissue to be modeled offers the closest developmental and epigenetic match. The ease or difficulty of obtaining such material varies widely, however, from the trivial (urine) to routine (skin scraping, blood) to different levels of invasive biopsies or surplus/waste surgery tissue [11]. Obtaining multiple different cell or tissue types from the same patient thus quickly becomes prohibitive. Slow culture growth and quick senescence (limiting cell numbers), as well as de‐differentiation outside the physiological environment (limiting cell function), are, moreover, characteristic of primary cells, and might even be intensified for disease phenotypes [17]. Some barriers, such as the intestines or lungs, also contain resident progenitor cells that can be proliferated and differentiated in vitro, but such populations are not ubiquitous, and derived‐cell function becomes dependent on protocol optimization. Alternatively, induced pluripotent stem cells (iPSCs)—reprogrammed from easily accessed patient blood or skin cells—have emerged since 2007 as a powerful, highly proliferative cell source for complex disease modeling. Numerous protocols were developed for differentiating iPSCs into specific cell types, or more accurately into cells that match the biological signature of corresponding primary cells or in vivo function. The preferred terminology is thus, for example, iPSC‐derived astrocyte‐*like* cells, but for simplicity we will shorten this to, for example, iPSC‐astrocytes. A critical challenge with iPSCs is representing age‐related and cumulative environmental influences, as derived cells often show immature or fetal expression patterns [11]. iPSCs may also retain epigenetic memory linked to the donor cell or its reprogramming, which may, for example, create bias toward/against certain differentiation lineages even between iPSC lines from the same donor, and can supersede disease‐specific genetic backgrounds [18, 19]. Deriving cells from iPSCs still offers tremendous opportunities where complex multicellular ensembles are concerned [11]. Both scenarios appear throughout this review, depending on the availability and proliferation of primary cells and the state of iPSC differentiation protocol development; a broad overview of this is shown in Table 1.

**TABLE 1:** Overview of the tissue barriers reviewed.

Barrier tissue	Barrier type^a^	Primary models^b^	iPSC models^c^	OoC models^d^
**3.1** **Blood and lymphatic systems**				
Blood vessels	Endothelial	o	+	Level 3.5
Lymph vessels	Endothelial	o	o	Level 2
Bone marrow	Endothelial	o	—	Level 3
Thymus	Epithelial	—	o	Level 0
Spleen	Endothelial	—	—	Level 0
Lymph nodes	Endothelial	o	—	Level 0
**3.2** **Central nervous system**				
Blood–brain barrier	Endothelial	—	+	Level 3.5
Blood–CSF barrier	Endothelial & Epithelial	—	o	Level 2
**3.3** **Skin**	Epithelial	+	+	Level 3
**3.4** **Ocular barriers**				
Outer blood–retinal barrier	Endothelial & Epithelial	—	+	Level 2.5
Inner blood–retinal barrier	Endothelial	—	—	Level 2
Cornea	Epithelial	—	+	Level 1–2
Anterior cavity	Epithelial	o	o	Level 0
**3.5** **Aural barriers**				
Blood–labyrinth barrier	Endothelial	—	—	Level 2.5
Other	Epithelial	—	o	Level 0
**3.6** **Respiratory system**				
Nasal mucosa	Epithelial	+	—	Level 2
Tracheobronchial tree and lung	Epithelial	o	+	Level 3
**3.7** **Gastrointestinal system**				
Oral mucosa	Epithelial	+	—	Level 1
Esophagus	Epithelial	o	+	Level 3
Stomach and colon	Epithelial	+	+	Level 2.5
Intestine	Epithelial	+	+	Level 3.5
Biliary tract	Endothelial & Epithelial	+	+	Level 3
Pancreas	Epithelial	—	+	Level 3.5
**3.8** **Urinary Tract**				
Glomerulus	Endothelial & Epithelial	+	+	Level 3
Renal tubule	Epithelial	+	o	Level 2.5
Bladder and associated ducts	Epithelial	o	o	Level 2
**3.9** **Reproductive system**				
Testes	Epithelial	—	o	Level 0
Prostate	Epithelial	o	o	Level 1
Other male glands	Epithelial	—	—	Level 0
Ovaries and fallopian Tube	Epithelial	—	+	Level 0
Uterus	Epithelial	+	+	Level 1–2
Fetal–maternal interface	Endothelial & Epithelial	—	o^e^	Level 2
Vagina and cervix	Epithelial	+	—	Level 2
Mammary glands	Epithelial	+	+	Level 1–2
**3.10** **Thyroid**	Epithelial	o	+	Level 0

*Note*: This table indicates the endothelial or epithelial nature of the barrier, the availability and ease of use of primary human cells, the degree of protocol development for iPSC‐derived differentiations toward barrier cell types, and the level of progress in OoC models. Together, these categories—though some of the classification is necessarily subjective—illustrate the varied progress across OoC barrier systems, from basic cell availability to advanced personalized disease modeling.

^a^ While most epithelia are also reliant on interactions with nearby blood and/or lymph vessels, we highlight this in the table only when the respective endothelium is notably specialized.

^b^ + easily accessible, highly proliferative, and well characterized; o partial or intermediate availability; – highly invasive, low yield, limited characterization or proliferation.

^c^ + protocols established and validated for most relevant cell types; o partial or intermediate availability; – no or minimal development.

^d^ Level 0: no reported human OoC; Level 1: cell line‐based OoC; Level 2: healthy‐genotypic primary/iPSC‐based OoC (2.5 – isogenic); Level 3: disease‐genotypic OoC (3.5 – isogenic).

^e^ Although respective protocols have been developed, the fetal‐maternal interface poses unique challenges for iPSC‐based modeling as discussed in the relevant Section.

Abbreviation: OoCc, organ‐on‐chip.

## 3 Biological Barriers and Their Models

### 3.1 Blood and Lymphatic Systems

#### 3.1.1 Blood Vessels

Blood vessel networks with arteries, veins, and capillaries, which differ in mechanical properties, compositions, and diameters, form a hierarchical transport system across the body. All vessel types share an innermost monolayer of endothelial cells (ECs; resting on a thin basement membrane), which governs vessel permeability. Some organs and their functions rely on leaky (e.g., kidney) or extremely well‐regulated (e.g., brain) vascular barriers, to be discussed in their own separate sections [20]; here, we focus on the predominant vasculature with tight inter‐cell junctions. Single‐layer EC capillary walls are often supported by interaction with pericytes (PCs), with coverage varying from 10% to 50% across organs [21]. Stronger and more elastic multi‐layer walls, containing elastin, collagen, and smooth muscle cells (SMCs), provide distensibility and mechanical strength and allow arteries to sustain blood pressure surges. Additional stability in these larger vessels is provided by an outermost layer of primarily collagen and fibroblasts. Angiogenesis and the maintenance of the microvasculature rely on the interaction of ECs with mural cells (MCs; i.e., PCs or SMCs), as well as on biomechanical stimuli from blood flow and pressure. ECs and PCs exist as heterogeneous and tissue‐specific populations, with phenotypes adapted to organ and vessel structure, blood derivatives, gas exchange, and metabolic requirements [22]. Biomechanical signaling is also influenced by tissue‐specific variations in shear (primarily borne by ECs) and pressure levels and cycles (primarily borne by SMCs) [23]. These interdependencies are especially evident in cardiovascular diseases, where deficits in extracellular matrix and SMCs can lead to vessel wall thinning and weakening, resulting in dilations and aneurysms, while pathological stiffening, lipid accumulation, and plaque formation are clinical manifestations of atherosclerosis [20, 24].

One of the most frequently used EC sources are human umbilical vein endothelial cells (HUVECs) isolated from donated umbilical cords [25]. Other regional ECs, as well as MCs, can also be obtained from less‐available surgical tissue [26, 27]. Major heterogeneity of phenotypes across different segments of the vasculature complicates the isolation of relevant cells, and availability of cells for in vitro models can be further restricted by quick senescence and low proliferative capacity [17, 22, 28]. One potential alternative is the use of SMC progenitors and EC progenitors that are naturally present in blood [22]. However, their origins and cellular identities remain subjects of ongoing scientific debate, and their low abundance in peripheral blood, along with a tendency to lose cell integrity, limits their suitability for expansion and long‐term studies. iPSC‐EC differentiation has shown great promise, with a broad range of protocols capable of establishing the cellular and functional characteristics of mature ECs [29]. MC‐like differentiation protocols are perhaps lagging behind, but lineage‐specific iPSC‐SMCs have been published [30]. Specific iPSC‐PC protocols are also emerging, such as those adapted from brain PC differentiations for the derivation of cardiac microvasculature, using strategies aimed at preventing commitment to an SMC fate [31]. Both iPSC‐ECs and iPSC‐MCs have since been applied for disease modeling, notably by Kelleher et al. toward the most common form of vascular dementia and genetic stroke (clinically affecting small arteries by, e.g., SMC degeneration) [32]. Patient or control iPSC‐ECs co‐cultured in a Transwell with patient iPSC‐MCs showed decreased survival and angiogenesis compared to cultures with control iPSC‐MCs, highlighting the disease‐relevant importance of paracrine signaling. The lack of angiogenic paracrine factors could be compensated by supplementation with the angiogenic growth factor VEGF, which rescued capillary structures. Given the relevance of blood vessel permeability and angiogenesis in tumor microenvironments, there have naturally been numerous efforts in this direction—including with OoCs and clinical tumor‐derived cells—but are considered outside the scope of our review [33].

An early example of iPSC‐based OoCs for analyzing rare genetic vascular disorders is found with Hutchinson–Gilford progeria syndrome (HGPS), which causes premature vascular aging and early cardiovascular failure. Patient‐derived iPSCs could even provide a valuable model for recapitulating aspects of general aging in an accelerated time frame. Atchison et al. developed a relevant 3D arteriole‐scale OoC by embedding patient‐derived iPSC‐SMC in a (somewhat unusually, freestanding) needle‐templated hydrogel tube and combining them with healthy human cord blood‐derived EC progenitors [17]. In line with vascular stiffening observed in patients, the researchers reported increased calcification, apoptosis, and thickness of the sub‐endothelial vessel layer along with decreased vasoactivity with patient‐derived iPSC‐SMCs (vs. healthy) after 4 weeks of culture. Replacing the primary ECs with isogenic patient‐derived iPSC‐ECs, the researchers subsequently found that EC expression of flow‐responsive genes was impaired and the engineered vessels exhibited EC dysfunction (vs. isogenic healthy), including markers of cardiovascular disease and compromised vasodilation and vasoconstriction [34]. This was in contrast to a discordant co‐culture of healthy iPSC‐SMCs and HGPS iPSC‐ECs, which demonstrated only diminished vessel relaxation, uniquely illustrating the distinct contributions of SMCs, ECs, and their crosstalk in the disorder. Ribas et al. also highlighted further opportunities afforded by OoC in this context, finding that HGPS iPSC‐SMC monocultures exhibited higher sensitivity to cyclic mechanical stretch and that this increased inflammatory profile could be alleviated by pharmacological intervention [23]. The additional importance of regional specification of iPSC‐SMCs for such studies was recently demonstrated by Liu et al. in a similar OoC (albeit with healthy cells) in terms of differential sensitivity to tensile stress [28].

Atherosclerosis is characterized by plaque formation due to lipid accumulation in the artery walls, followed by an inflammatory response, and can have both genetic and lifestyle‐related causes. During the initial stages of atherosclerosis, macrophages infiltrate the endothelial layer, where they contribute to inflammation, foam cell formation (i.e., lipid‐laden macrophages and SMCs), and plaque formation, ultimately setting the stage for blood clot formation, known as thrombosis. To study this cellular crosstalk, Middelkamp et al. embedded lipid‐laden macrophages in their iPSC‐derived 3D vessel OoC [35]. They relied on a more typical, chip‐enclosed collagen I hydrogel with a circular lumen, and sequentially seeded macrophages and iPSC‐ECs. A human whole blood perfusion assay showed increased deposition of fibrin when lipid‐laden macrophages were included compared to controls lacking them. Maringanti et al. subsequently employed an OoC with five similar 3D vessels in parallel to further delve into this foam cell crosstalk [36]. Their OoC comprised HUVECs and primary vascular SMCs, with lipid‐laden macrophages optionally admixed to the SMCs to resemble early‐stage human atherosclerotic lesions. Inclusion of the foam cells led to elevated expression of atherosclerosis‐associated markers as well as increased extravasation and recruitment of circulating monocytes. The researchers also noted pathophysiological migratory behavior of the SMCs under flow in the atherosclerotic condition, a key OoC advantage. Zhang et al. modeled earlier‐stage processes in another comparable OoC based on primary ECs and surrounded by hydrogel‐embedded primary SMCs and fibroblasts [37]. They revealed that already the exposure to a low‐density lipoprotein decreased vasoactivity and induced an inflammatory environment, including tissue accumulation, activation, and foam cell formation from circulating primary monocytes. After lipoprotein removal, the engineered vessels partially recovered from this early atherosclerosis‐like phenotype, though increased vessel permeability and foam cells remained.

**FIGURE 2:**
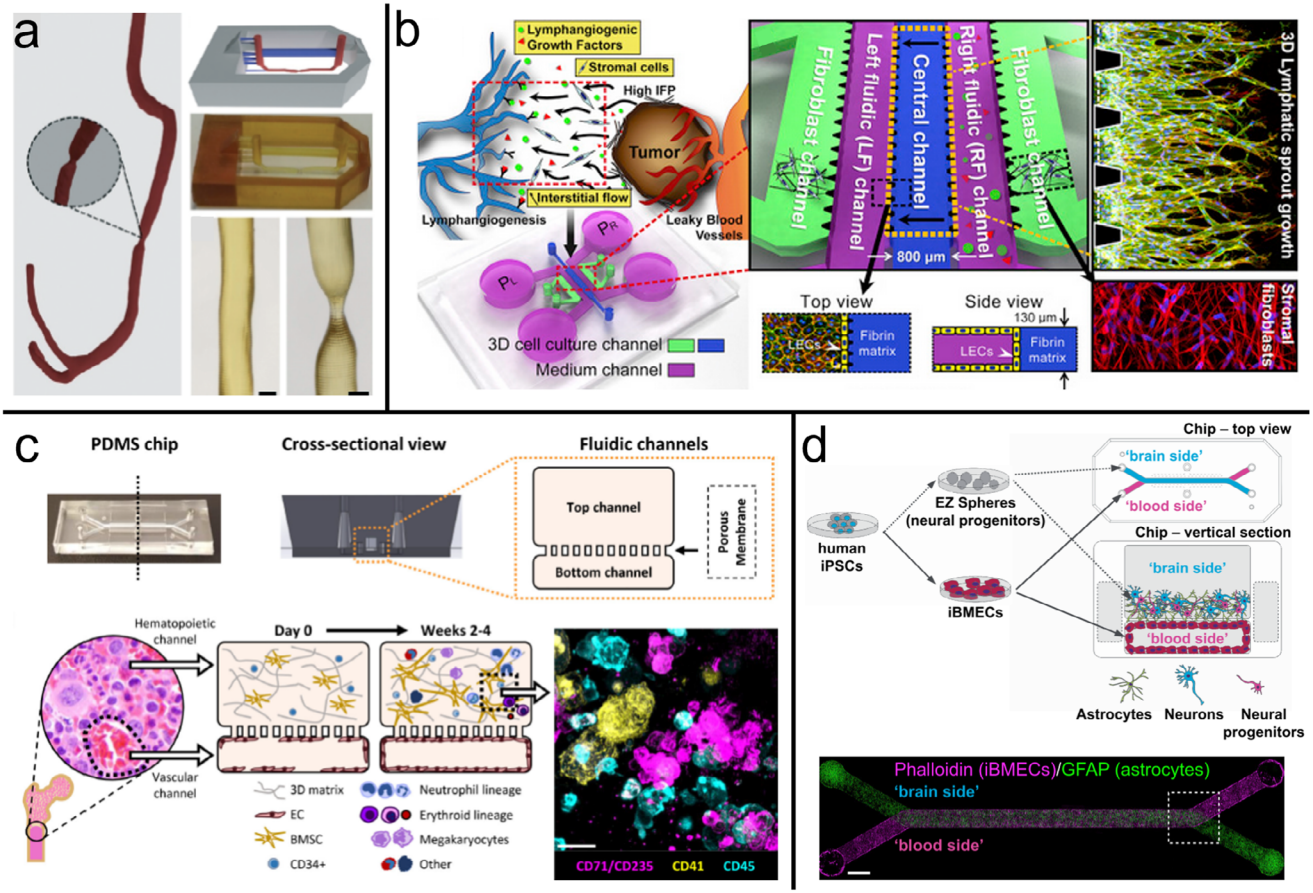
Endothelial barriers‐on‐chips. (a) Blood vessel OoC. Stenotic (and healthy) coronary artery segments were derived from computed tomography angiography and reproduced in an OoC using 3D printing to closely replicate physiological flow profiles for the resident endothelium. Adapted under the terms of the CC BY license [40]. Copyright 2017, Costa et al. (b) Lymphatic vessel OoC. The left and right fluidic channels (purple) allowed to adjust flow and biochemical factors for directional stimulation of lymphatic endothelium (LECs; yellow) seeded on a fibrin matrix (blue) to study lymphangiogenesis in this lateral/hydrogel OoC. Adapted with permission [42]. Copyright 2016, Elsevier. (c) Human bone marrow OoC. Incorporation of hematopoietic progenitors and bone marrow stromal cells (BMSC) in a 3D matrix above a bottom channel lined by endothelium (EC)s facilitated hematopoiesis in this commercial vertical/membrane chip. Adapted with permission [43]. Copyright 2020, Springer Nature. (d) Blood–brain barrier (BBB)‐OoC. Fully isogenic model with BBB iPSC‐endothelium (iBMECs; purple) and a heterogeneous population derived from iPSC‐neural progenitors inside a commercial vertical/membrane chip, employed to study two genetically‐driven disorders. Adapted with permission [44]. Copyright 2019, Elsevier. iPSC, induced pluripotent stem cell; OoC, organ‐on‐chip.

Given that thrombotic complications often follow EC dysfunction in atherosclerosis, several OoC studies have specifically focused on modeling thrombus formation under inflammatory or flow‐altered conditions. Manz et al. proposed to study and visualize thrombus dynamics by perfusion of whole blood through straight or constricted (i.e., stenotic‐like) microfluidics seeded with primary ECs [8]. They were able to show increased thrombogenic properties for pulmonary arterial ECs from a patient with chronic thromboembolic disease. Mathur et al. also leveraged an OoC with a single microfluidic channel for thromboinflammatory research [38]. Employing blood outgrowth ECs (i.e., derived from circulating EC progenitors), their model resembled mature ECs in terms of sensitivity to shear, growth pattern, morphologies, and expression of adhesion markers. The OoC exhibited physiologically relevant cytokine‐induced inflammatory responses resulting in platelet hyperactivity and induction of thrombus formation. Other works (resorting, however, to HUVECs) have focused further on the impact of vascular geometry and flow on thrombogenesis, successfully mimicking pathophysiological conditions in OoCs designed to mimic high‐risk venous valve cusps [39], or directly resembling patient‐specific clinical thoracic scans via 3D printed microfluidics (Figure 2a) [40]. More recently, Cuartas‐Vélez et al. showed how visible‐light optical coherence tomography can capture the temporal evolution of thrombi formation in their HUVEC‐based vessel OoC [41].

Another condition closely linked to atherosclerosis is abdominal aortic aneurysm. Aortic aneurysm is a condition in which the wall of the aorta weakens and becomes abnormally enlarged, which can occur in different regions of the body. Paloschi et al. analyzed how hemodynamic forces contribute to abdominal aortic aneurysm by using an OoC designed to mimic medium‐to‐large arteries [45]. Primary human aortic ECs and SMCs were co‐cultured on opposite sides of a vertical/membrane OoC. They found that high‐shear laminar flow, as characteristic of healthy arteries, yielded gene expression patterns resembling the same in vivo, while static or low‐shear oscillatory conditions resembled diseased artery segments. They further proceeded to co‐culture healthy ECs with SMCs from abdominal aortic aneurysm disease patients. They observed that administration of a drug in the vascular compartment led to downregulation of angiogenesis and inflammation markers in the ECs, as well as a shift toward a contractile phenotype in the SMCs, reflecting protective effects observed in vivo.

Overall, combining these precisely adjusted vessel geometries and flow patterns with relevant patient‐derived vascular cells and blood components offers opportunities for detailed investigation of healthy and pathological states and interactions at vessel walls.

#### 3.1.2 Lymph Vessels

In addition to blood vessels, the second extensive body‐spanning vascular network is the lymphatic network that is responsible for transporting lymph, a fluid containing excess blood plasma, nutrients, antigens, and immune cells. Initial lymphatic capillaries consist of ECs connected by button‐like, discontinuous tight junctions, which facilitate entry of fluid and solutes from interstitial spaces of tissues. In contrast, collecting lymphatic vessels show zipper‐like, continuous tight junctions between ECs that minimize lymph leakage. These vessels also have characteristic internal valves and are surrounded by lymphatic SMCs, both of which contribute to the unidirectional propulsion of lymph flow [10, 33]. While lymphatic ECs across different organs share common molecular signatures, observed heterogeneity suggests the existence of organ‐specific markers, which remain mostly unexplored [10].

Isolation of primary adult lymphatic ECs is possible from lymph nodes or skin, making them comparatively accessible, and at least one immortalized line has been generated [46]. Similarly, at least one iPSC differentiation protocol has resulted in pure cells of the lymphatic iPSC‐EC lineage, expressing lymphangiogenic factors and forming new vessels [47]. Compared to blood vessels, fewer studies tried to model the lymphatic system. Still, lymphatic dysfunctions, contributing to various diseases, including lymphedema, obesity, cardiovascular and inflammatory diseases, and cancer metastasis, have attracted interest [10]. Inclusion of patient‐derived lymphatic cells for personalized modeling does not appear to have been addressed yet.

Bringing lymphatic OoC into the context of disease models, a number of tumor‐lymphatic microfluidic models were built using primary or immortalized lymphatic ECs to study the interaction of primary tumors with their microenvironment [33, 48]. Highlighting the particular advantages of OoCs, Kim et al. found that interstitial flow acts as an important regulatory cue of lymphangiogenesis: initiation and outgrowth of lymphatic vessels were suppressed downstream of interstitial flow, whereas sprouting against the pressure gradient was augmented (Figure 2b) [42]. For back‐to‐back co‐cultures of lymphatic ECs and blood vessel ECs (both primary) in a vertical/membrane OoC, Sato et al. found that the characteristic barrier properties (tracer permeability and junction proteins) of both vessel types were enhanced by continuous or pulsating flow compared to static conditions [49]. Ilan et al. further evaluated the effects of different flow regimes and two vascular endothelial growth factors (VEGF‐A and VEGF‐C) on lymphatic EC junctions, sprouting, and cell contractility. Relying on needle‐templated microchannels in collagen I, they analyzed the responses of primary human lymphatic ECs to luminal flow, interstitial flow, a combination of both, or lack of flow [50]. Discontinuous EC junctions developed under interstitial flow combined with VEGF‐C, whereas lymphatic sprouting and zipper‐like junctions were observed with VEGF‐A under interstitial flow alone or when both flow types were combined, showcasing the possibilities for modeling distinct lymphatic vessel types in OoCs.

In the future, including different ECM compositions as well as lymphatic mural cells and immune cells in such OoCs can offer insight on lymphatic fluid homeostasis and immune responses [51]. Expanding on the applications beyond cancer metastasis, Kraus et al. have, for instance, proposed combining lymphatic OoCs with specialized connective tissue models to study chronic inflammatory joint diseases such as rheumatoid arthritis [52]. In this disorder, an initial expansion of the lymphatic system is followed by its abnormal contraction and collapse, ultimately impairing lymphatic drainage and immune cell clearance.

#### 3.1.3 Lymphoid Organs

Lymphoid organs and their specialized tissue architectures serve as sites of origin, maturation, and control for different blood cell types [43, 53, 54]. Red blood cells are mainly responsible for oxygen transport; white blood cells are the major cellular component of the multilayered defense of a human body, acting in conjunction with physical barriers and acellular components to establish an efficient innate and adaptive immune system [55]. In the hematopoietic compartment of the bone marrow, stem and progenitor cells proliferate, and interact with various stromal cells that support their differentiation [43]. Whereas most cells then enter the systemic circulation in a mature state, some white blood cell progenitors enter the thymus for T lineage commitment (again guided by stromal cell populations). The other lymphoid organs, including the spleen's white pulp and lymph nodes, serve as complex control points for adaptive immunity as they collect and filter antigens, antigen‐presenting cells, cytokines, and serum proteins from blood and lymph circulation, respectively [56]. The red pulp of the spleen, meanwhile, serves as a control point for red blood cells [54]. EC barriers play crucial roles in regulating these processes. Specialized vascular ECs mediate transmigration of hematopoietic cells into and out of the lymphoid organs. In bone marrow and spleen, this is closely linked to the maturation processes [57, 58]. At lymph nodes, the afferent lymphatic vessels transition into branched sinus systems [59]; their specialized lymphatic EC barrier separates naive adaptive immune cells and the immunogenic material carried by the lymph fluid, mediating their interactions. In the red pulp of the spleen, finally, vascular ECs feature a unique microstructure, with micrometer‐wide intracellular slits that strictly challenge the deformability of red blood cells. Stiffer cells, approaching the end of their life span of 100–120 days or demonstrating a pathological background, are sequestered and engulfed by macrophages that can detect reduced surface integrity [54, 60]. Around 1% of red blood cells are thus cleared from the circulation before they might block small vessels, resulting in organ damage.

Blood cells, including progenitors and immune cells that make up major fractions of lymphoid organs, can be received from a blood sample or minimally invasive liquid biopsy [61]. Primary tissue‐resident cell types are less accessible, with options ranging from bone marrow donation to biopsies and explants [62, 63]. Differentiation of thymic stromal cells from iPSCs recently achieved improved outcomes, with T cell crosstalk and 3D hydrogel chemistry identified as key aspects to consider for maturation [64]. iPSC protocols for the regionally specific ECs, however, remain under‐researched.

Limited access to the living bone marrow microenvironment makes investigating hematopoietic development and genetic mutations affecting this development challenging. To overcome these limitations, Chou et al. created a human bone marrow‐OoC, which allowed for differentiation and maturation of multiple blood cell lineages (Figure 2c) [43]. A 3D co‐culture of hematopoietic progenitor cells and bone‐marrow‐derived mesenchymal stromal cells (MSCs; from hip replacement surgery tissue) was embedded in fibrin gel inside the top channel of a commercial vertical/membrane chip. The bottom channel was lined by HUVECs and perfused with media to support both compartments. The OoC system improved oxygen delivery and enhanced in vitro hematopoiesis compared to static well‐plate culture. Notably, the inclusion of hematopoietic progenitors from patients of a rare genetic syndrome (arising from mutation in the SBDS gene) gave rise to hematopoietic defects that could also be observed with this disorder in vivo. Not only did the OoC recapitulate impaired progenitor maintenance and reduced numbers of developing cells, but it also led to the discovery of a previously unknown abnormality in the maturation of neutrophils. Building on these advancements, Aleman et al. developed a complementary OoC model to examine the interactions between hematopoietic progenitors and specific bone marrow niches [62]. They constructed the periarterial, perisinusoidal, mesenchymal, and osteoblastic niche by enclosing the respective primary (or, for osteoblasts, MSC‐differentiated) cells in hyaluronan/gelatin hydrogel, located in parallel chambers of a single recirculating OoC. This allowed them to study preferred migration and retention of circulating hematopoietic progenitors, recapitulating a murine in vivo finding that these cells home to three out of the four niches (i.e., all except the arterial). Going forward, this bone marrow niche‐focused system could potentially uncover niche‐specific contributions to disease pathology associated, for example, with the SBDS mutation encountered above.

The specialized microanatomy and circulation system of the human spleen differ substantially from rodent models, making OoCs all the more attractive [65]. Yet we could not identify any true OoC models of the spleen, though some researchers have studied the red blood cell filtration function in acellular microfluidic devices by employing engineered micro‐slits [54, 60, 66]. In a first attempt to recreate the complex lymph node microenvironment, Shanti et al. created a multi‐compartmentalized OoC [67]. The central elliptical chamber contained three concentric regions, separated by micropillars, with collagen‐embedded B cells and T cells placed in opposite halves of the innermost region. This design, which mimics the structural organization of a lymph node, enabled interactions between the immune cell types across the chamber boundaries. This setup allowed for quantification of cell–cell and cell‐antigen interactions, as well as the analysis of immune cell activation after antigen exposure. Although the flow patterns within the OoC successfully recapitulated fluid distribution observed in lymph nodes, the model lacked ECs.

Since a lack of compartmentalization in current model systems may impair replication of immune responses, like the ones taking place during immunotherapy, models that mimic the distinctly organized structure of lymphoid organs are needed [55]. During different physiological or pathological conditions, flow rates and direction can change (e.g., enhanced flow in inflammatory processes), which can influence the transport and interaction of immune cells and should thus also be considered in model design. The impact of specialized ECs on the initiation and progression of diseases also remains unexplored and could greatly benefit from investigation using OoCs.

### 3.2 Central Nervous System

#### 3.2.1 Blood–Brain Barrier (BBB)

The BBB is a selectively permeable barrier that regulates what can enter the brain from the bloodstream, consisting of three main cell types: brain microvascular ECs, PCs, and astrocytes [68]. Brain capillary ECs are particularly rich in tight junctions, prohibiting paracellular transport of most soluble compounds and restricting transport of small ions. Instead, most transport across the BBB is transcellular, and the metabolic function of the brain and its neurons (which consume most of the energy) is therefore heavily dependent on microvascular EC barrier integrity and transport mechanisms [69, 70]. As in other microvasculature, a surrounding layer of basement membrane and region‐specific PCs provide biomechanical stability and contractility. Lastly, astrocytes are a type of glial cell that can closely interact with brain microvasculature through astrocytic endfeet. Astrocytes play a regulatory role in the BBB, secreting various factors that modulate BBB permeability. While the cell types discussed generally form the core of BBB models, other cell types also contribute to neuro‐vascular coupling in vivo, including microglia (resident macrophage analogues, thus relevant, e.g., with injury or inflammation) and oligodendrocytes (supporting neuronal signal transduction, with precursor cells involved in angiogenesis) [68].

Isolation of any of these BBB cell types is difficult, generally relying on temporal lobe resections, as performed in intractable epilepsy patients. The yield of such procedures is typically low, and cell purity and proliferation are limited [71]. The first protocols for BBB iPSC‐EC differentiation were published a decade ago based on neural‐like co‐differentiation and adhesion‐based cell selection [72, 73], with variations and improvements to follow. However, the iPSC‐ECs derived through these protocols have been noted to express genes marking a partially epithelial nature [44, 74, 75]. It has been countered that these iPSC‐ECs possess both a BBB‐like passive permeability and BBB‐like active transport characteristics, and thus remain a suitable model for BBB ECs [76]. One proposed approach, proposed by Lu et al., is to enhance the endothelial characteristics of these cells by transcription factor overexpression using suitably engineered iPSC lines [77]. Another and more common strategy is to employ non‐BBB vascular iPSC‐EC [29, 78], where a recent preprint by Nogueira Pinto et al. showed that co‐culture with brain‐specific iPSC‐PCs, when combined with a chemical pathway modulator, significantly enhanced the BBB‐like transcriptomic signature of the ECs [79]. They also pointed at responsible transcription network pathways that could form the basis for relevant transcription factor overexpression in the future (employed with iPSC‐ECs already toward enhancing protocol efficiency) [80]. Barrier integrity, at least in terms of TEER, still falls short of in vivo estimates using either of these two strategies, and neither can match the simplicity of small‐molecule differentiation protocols.

Various protocols have been published to derive also PCs and astrocytes from iPSCs [81]. For both cell types—though more so for PCs—recapitulation of in vivo functional and regional heterogeneity remains an open question, and comparatively long iPSC‐astrocyte differentiation times (given a corresponding late in vivo maturation) can pose practical challenges for personalized disease models.

The first multicellular iPSC‐derived BBB‐OoC was demonstrated by Vatine et al. in a commercial vertical/membrane chip (Figure 2d) [44]. BBB iPSC‐ECs were co‐cultured with iPSC‐neural progenitors, resulting in a barrier that displayed physiologically relevant integrity/permeability, which could be maintained also for whole blood perfusion. They showed that physiological shear resulted in upregulation of a range of junction protein expression (distinct from both static and low‐flow culture) as well as BBB‐specific transporters. Moreover, the researchers demonstrated the viability of their BBB‐OoC as a personalized model for two genetic disorders. First, monocarboxylate transporter 8 deficiency, a form of psychomotor retardation arising from mutation in this thyroid hormone transporter protein. In line with their hypothesis, BBB‐OoC from such patients (or with an induced mutation) were found to be significantly less permeable to thyroid hormone compared to models derived from control iPSCs (including gene‐corrected patients). Second, the biologically somewhat more complex Huntington's disease. Here, a patient‐derived OoC showed higher tracer permeability compared to healthy controls, recapturing the compromised BBB of Huntington's patients. Targeting the same disease, Linville et al. constructed a BBB‐OoC from only brain iPSC‐ECs, derived from juvenile‐onset Huntington's patients. They employed needle‐templated 3D collagen tubes to better mimic the vascular geometry compared to a flat membrane [82]. Their Huntington's‐genotypic OoC exhibited identical small‐molecule permeability to a gene‐corrected control. However, they reported a reduction in both cell loss and proliferation of the diseased iPSC‐ECs, suggesting changes in endothelial turnover. Furthermore, the patient‐derived vessels were more prone to adhesion by a perfused monocyte‐like immune cell line despite the lack of exogenous inflammatory stimulation.

Expanding the use of BBB‐OoCs to other disorders, Lall et al. endeavored to build a BBB‐like model of amyotrophic lateral sclerosis (ALS) to study the functional characteristics of the diseased cells [83]. ALS is an adult‐onset neurodegenerative disease that causes the progressive loss of motor neurons until death (usually respiratory failure within 2–5 years). Like Vatine et al. earlier, Lall et al. leveraged a commercial vertical/membrane chip with BBB iPSC‐ECs (from one healthy control), but with spinal motor iPSC‐neurons (non‐isogenic; from ALS patients or controls) in the second compartment. Performing bulk transcriptomic and proteomic analysis, they were able to identify a clear early ALS phenotype centered around synaptic plasticity and glutamate signaling. This was in contrast to well‐plate cultures, and in spite of the lack of known (i.e., screenable) ALS genetic mutations in the patients, indicating the promise of OoCs as predictive models. The authors were, however, unable to observe neurodegeneration, noting the limitation of iPSC‐neurons for adult‐onset diseases. Lastly, Brown et al. developed an iPSC‐derived BBB‐OoC to study tuberous sclerosis complex, a genetic disorder that leads to the development of non‐cancerous tumors in different organs, including the brain [84]. Their model consisted of a BBB iPSC‐EC vascular channel on top of a vertical/membrane chip, with iPSC‐astrocytes on the opposing side, along with a synthetic hydrogel hosting glutamatergic iPSC‐neurons. They demonstrated that BBB permeability increased in the patient‐derived OoC compared to healthy controls—an effect not observable from equivalent Transwells (though the clinical relevance of BBB permeability in the disorder is an open question). Crucially, the researchers also demonstrated a recovery of BBB integrity to baseline in a discordant model when replacing only the iPSC‐astrocytes with healthy controls. This broadly aligns prior insights from murine models and highlights the unique opportunities afforded by iPSC‐based OoCs to study individual cellular contributions to disorders.

Beyond these truly genotypic disease models, Pediaditakis et al., for instance, developed an OoC model of α‐synuclein accumulation, a process that plays a central role in synucleinopathies such as Parkinson's disease [85]. Aiming to mimic the substantia nigra BBB in a commercial vertical/membrane chip, they seeded its opposing membrane sides with BBB iPSC‐EC and with a mixture of primary human astrocytes, microglia, and PCs, as well as dopaminergic iPSC‐neurons. Addition of fibrillar α‐synuclein was shown to lead to neuronal apoptosis, an increase in BBB permeability, and changes in iPSC‐EC genetic expression. Treatment with the disaccharide trehalose was shown to ameliorate BBB degradation and brain inflammation, mimicking prior studies in mice. Using instead primary brain ECs alongside iPSC‐astrocytes and iPSC‐neurons, Wevers et al. presented a model for ischemic stroke [86]. Cells were cultured on opposing sides of a collagen gel in a commercial lateral/hydrogel chip, with the BBB subjected to simultaneous hypoglycemia, chemical hypoxia, and halted perfusion. This resulted in a compromised barrier and a significantly disrupted energy metabolism across the BBB, whereas any individual intervention only elicited partial effects, showcasing specific OoC advantages over static culture. McCloskey et al., meanwhile, combined an iPSC‐BBB‐OoC with circulating primary neutrophils to study inflammatory processes [87]. Employing an ultrathin (100 nm) membrane with iPSC‐ECs and iPSC‐PCs on opposing sides, they could show that neutrophil infiltration was reduced in the presence of iPSC‐PCs compared to monoculture when they applied a cytokine cocktail vascularly (sepsis), but not perivascularly (neuroinflammation).

The BBB is essential for maintaining the health of the brain, and its malfunction is part of, or lies at the root of, many brain‐affecting diseases, such as Huntington's, Parkinson's, or Alzheimer's. Furthermore, the BBB presents an obstacle for drug delivery to the brain. For these reasons, in vitro models of the BBB, such as BBB‐OoCs, can be useful tools to dissect relevant pathological and pharmacological questions. For the purpose of personalized models, iPSCs represent the best way forward due to the inherent limitations in harvesting and maintaining primary BBB cells. Yet the lack of reliable markers for certain cell types (e.g., brain PCs) makes it difficult to judge the product of current differentiation protocols. This underscores the need for further characterization of primary human cells.

#### 3.2.2 Blood–Cerebrospinal Fluid Barriers

Besides blood circulation, the brain is home to another compartmentalized fluid system: the cerebrospinal fluid (CSF), produced largely in the choroid plexus of each brain ventricle through blood filtration [88]. CSF flows through the glymphatic system, which is somewhat analogous to the lymphatic system, in that it carries metabolic waste of the central nervous system away and helps maintain homeostasis. In contrast to lymph, glymphatic CSF largely flows through periarterial and perivenous spaces rather than separate vessels. It also acts as a protective layer between the brain and the skull and can house immune cells. In the choroid plexus, the blood–CSF barrier is formed by the close interaction of fenestrated ECs with a cuboidal epithelium, which possesses villi on the apical side [89]. Exchange of compounds across the epithelium is highly regulated through tight junctions, active transport processes, and metabolic enzymes to regulate CSF homeostasis [90]. The blood–CSF barrier, moreover, includes the arachnoid mater, which encases the entire brain [91, 92]. This is made up of arachnoid barrier cells, a type of epithelial‐like cell with tight junctions. At arachnoid granulations, these form an interface with the venous system of the dura mater, where the CSF gets reabsorbed [93].

At least two protocols have been described for the generation of iPSC‐derived choroid plexus organoids, with one demonstrated to produce CSF‐like fluid [94, 95]. Both research groups employed their organoids to model COVID‐19 infection, showing that the virus can compromise the blood‐CSF barrier, a possible contribution to its central nervous system symptoms [96]. Yet Zhou et al., in developing one of the first human choroid plexus OoCs, chose to rely on primary cells [97]. Co‐culturing brain microvascular ECs and choroid plexus epithelial cells on opposing sides of a vertical/membrane chip, they demonstrated improved barrier function and upregulation of a variety of genetic pathways, including selective transporters, compared to either static or mono‐cultures. For arachnoid modeling, meanwhile, Endres et al. built a Transwell co‐culture system using brain iPSC‐ECs and primary arachnoid cells (obtained from surgical tumor resections) to study meningococcal infection [98]. While informative, this type of system lacks the dynamic flow and complexity of chip‐based models.

Despite the critical role of the blood–CSF barrier in brain homeostasis, few human‐relevant in vitro models exist to study its structure and function in detail. The availability of iPSC differentiation protocols, particularly for arachnoid cells, remains a limitation. Yet personalized blood–CSF barrier OoCs would be attractive due to the direct role of the barrier in syndromic hydrocephalus, in addition to being implicated in disorders like Alzheimer's, multiple sclerosis, schizophrenia, or Huntington's [88].

### 3.3 Skin

The skin represents the body's outermost barrier. As the biggest multifunctional human organ, it offers protection against various environmental risk factors such as toxic or infectious substances, mechanical injuries, UV light and temperature changes, and balances water release from inside the body. The outermost layer (epidermis) consists predominantly of epithelial keratinocytes; they not only form a physical barrier for potential harms, but they can also take part in activating the innate immune system and presenting antigens to T cells [99]. The underlying dermis is primarily made up of fibroblasts, which synthesize the ECM and secrete factors that can promote proliferation and differentiation of keratinocytes. Besides T cells, this layer accommodates a range of immune cells, including macrophages, dendritic cells, and mast cells [99]. Like all external epithelia of the human body, there is also a complex and variable population of bacteria colonizing the outer surface that plays a role in skin homeostasis [100].

Epidermal models built solely based on keratinocytes can be sufficient for analyzing the skin's main barrier function, which is affected in many cutaneous disorders [99, 101]. Primary keratinocytes are relatively accessible from skin biopsies [102]. Fibroblasts are similarly accessible and, moreover, notably proliferative; alongside exogenous and/or secreted ECM, they can resemble the dermis [99]. Layered in a full‐thickness skin equivalent, the crosstalk between keratinocytes and fibroblasts alone can improve keratinocyte phenotypes [103]. Other cell types of the dermis and hypodermis, in particular immune cells, are typically less accessible from patients, although some may be gained as lipoaspirate from surgeries [99, 102].

To circumvent these limitations, directed differentiation of iPSCs into various skin‐residing cell types was pursued, including iPSC‐keratinocytes and iPSC‐fibroblasts as well as iPSC‐melanocytes, iPSC‐cutaneous neurons, iPSC‐adipocytes, and even cell types involved in hair follicle and gland development [101]. Such efforts have already shown promise in modeling genetic disorders, for instance, with an ultra‐rare point mutation visible as destructive premature aging in children. Patient‐derived iPSCs reached the keratinocyte lineage faster compared to control iPSCs, lending weight to a stem cell depletion hypothesis for the disorder [104]. Besides cellular complexity, natural mechanical stimuli like shear stress and compression have been shown to improve epidermal morphogenesis and maturation, for example, in terms of protein expression, arrangement, and barrier function [3, 105]. Skin‐OoC models thus have great potential for precise and personalized outcomes, analyzing autoimmune reactions, allergies, inflammation, edema, and wound healing, as well as chronic inflammatory skin diseases like psoriasis and atopic dermatitis [3, 99].

The dermis of hidradenitis suppurativa patients exhibits keratinized epithelial tunnels accompanied by adjacent tertiary lymphoid structures. Within these structures, proliferative T and B cells producing antibodies reactive to keratinocytes were identified as drivers of the chronic skin inflammation [106]. Yu et al. developed a chip to resemble the disease‐determining lymphoid structures: a central area, designated for B and T cells, was initially filled with a hydrogel solution containing sacrificial gelatin, while primary human skin fibroblasts diluted in Matrigel were seeded into a surrounding inner ring area [106]. Following gelation of the Matrigel and removal of the gelatin, fluorescently labeled B and T cells were introduced into the central compartment. Incorporating patient‐derived fibroblasts into the OoC revealed how these cells enhance lymphocyte aggregation, and an initial disruption of these feedback loops between fibroblasts and B and T cells effectively interfered with initiation of lymphocyte aggregation. This deduction of a novel treatment avenue might be transferable to other autoimmune diseases. Matei et al. instead created a full‐thickness skin equivalent with keratinocytes on top of decellularized pig intestine, revascularized with ECs and seeded with fibroblasts (all human primary cells) [107]. The millifluidic OoC facilitated physiological pulsatile perfusion and was compatible with both submerged and skin‐air interface culture. The importance of cellular crosstalk could be demonstrated by vascular induction—and treatment—of skin fibrosis.

Other researchers have pointed out further OoC possibilities to study the aforementioned autoimmune disorders of the skin. Ren et al., for instance, established controlled gradients of psoriasis‐relevant chemokines in a lateral/hydrogel OoC to study (healthy‐donor) T cell homing and migration across epithelial (immortalized keratinocytes) and/or endothelial (HUVECs) barriers in either direction [108]. In their induced inflammatory environment, they were able to show that a specifically engineered protein treatment could decrease the chemokine–receptor‐mediated T cell migration. Kim et al., meanwhile, induced atopic dermatitis in a skin‐OoC with interleukin (IL)‐4 and IL‐13, characteristic of the disorder's T helper 2 cell activation [109]. Their gravity‐perfused skin model, consisting of healthy primary keratinocytes and fibroblasts, replicated pathological features like decreased expression of the epidermal structural protein filaggrin. They also observed dermatitis‐like uneven and rough outer skin layer topography, depending on the applied IL concentration. To also capture the disease's neuroimmune interactions, innervated atopic dermatitis models were proposed. Notably, Lightfoot Vidal et al. demonstrated patient‐specific inflammatory response in a static, off‐chip model by integrating reprogrammed neural stem cells with an inner human skin layer from patient lipoaspirate (yielding high cellular complexity and immunocompetence) and middle/outer layers from hydrogel‐embedded primary fibroblasts and keratinocytes [110].

**FIGURE 3:**
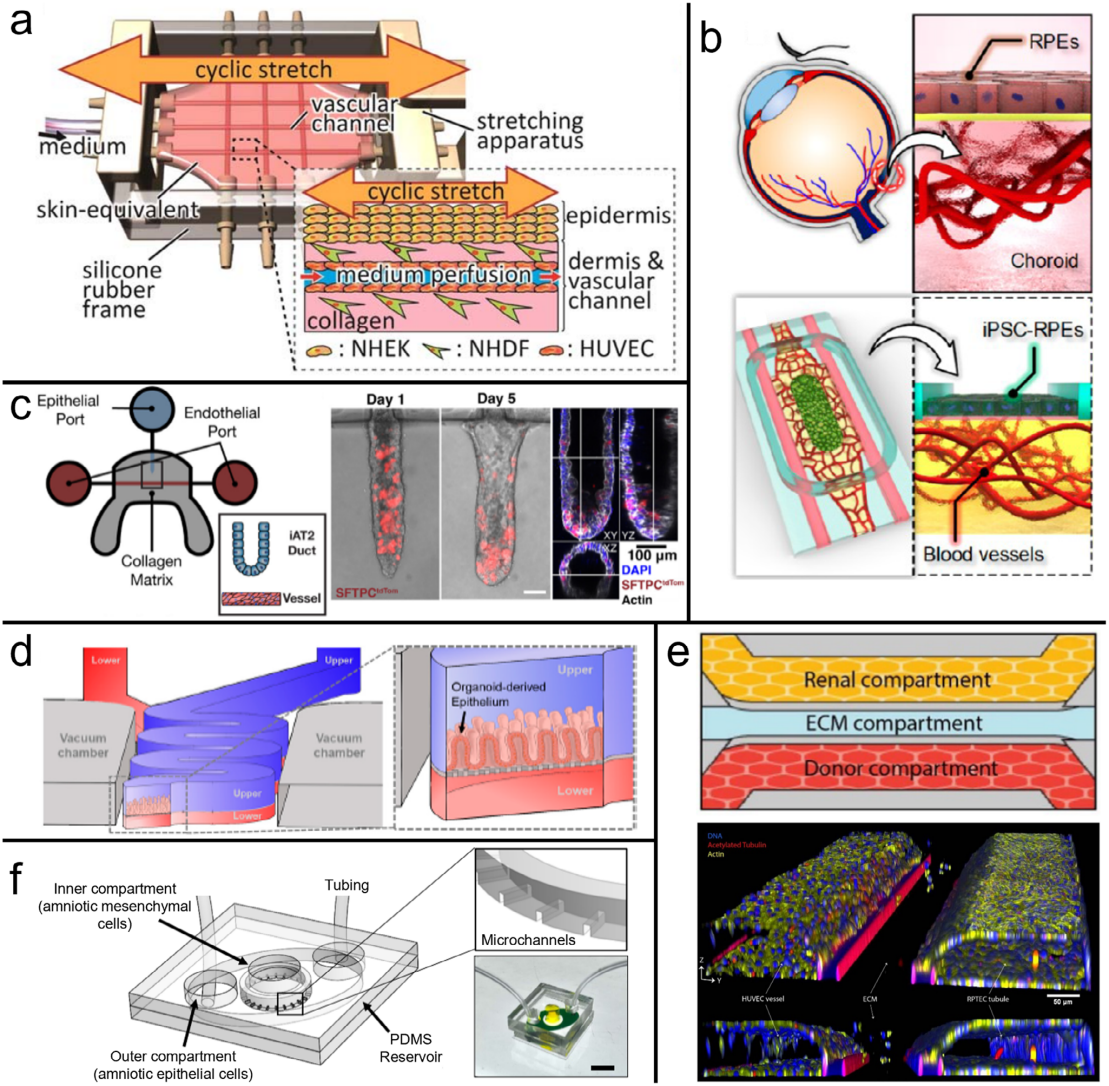
Epithelial Barriers‐on‐Chips. (a) Skin OoC. Needle‐templated endothelialized (HUVEC) vascular channels facilitate perfusion in a fibroblast (NHDF)‐seeded hydrogel dermis underlying the epidermal keratinocytes (NHEK), with a stepper motor imposing cyclic stretch to the 3D‐printed chip. Adapted with permission [114]. Copyright 2019, IOP Publishing. (b) Outer blood–retinal barrier OoC. Retinal epithelium (iPSC‐RPEs) forms a dense monolayer on top of a hydrogel hosting self‐assembled perfusable choroidal microvasculature. Adapted with permission [116]. Copyright 2019, ACS. (c) Alveolus OoC. Inside a collagen matrix, a blind‐ended channel is lined with lung epithelium (iPSC‐AT2), and a perfusable vascular channel lined with endothelium. Adapted under the terms of the CC BY‐NC license [117]. Copyright 2024, Gagnon et al. (d) Intestine OoC. Schematic illustration of the vertical/membrane OoC, with the upper channel (blue) resembling the intestinal mucosal interface based on dissociated primary organoid cells. The tortuous channel geometry helped establish physiodynamic multiaxial cell stretch (imposed by vacuum‐channel actuation) and nonlinear hydrodynamic shear. Adapted under the terms of the CC BY license [118]. Copyright 2020, Shin et al. (e) Proximal renal tube OoC. Schematic and confocal reconstruction of a commercial lateral/hydrogel chip with a proximal tubule epithelium‐lined renal channel (yellow) adjacent to collagen (ECM; blue). The endothelialized vascular channel (“donor,” red) was also used to introduce monocytes of different donors as an immune component. Adapted under the terms of the CC BY license [119]. Copyright 2025, Gijzen et al. (f) Amnion OoC. The (perfused) outer chamber with amniotic epithelial cells and the inner (static) chamber with amniotic mesenchymal cells are separated by collagen‐filled microchannels in this lateral/hydrogel chip. Adapted with permission [120]. Copyright 2024, Springer Nature.

Mechanical forces are fundamental to maintaining skin homeostasis and can contribute to the onset and progression of skin diseases. For example, the most common hereditary skin blistering disorder, epidermolysis bullosa simplex, arises from mutations affecting structural, cell–cell, and cell–matrix adhesion proteins, compromising mechanical integrity. Consequently, even low‐intensity mechanical stress results in intraepidermal cleavage and skin blistering of variable severity [111]. Where OoC studies integrate biomechanical stimulation, however, the focus remains on aging and wrinkling [112]. Lim et al., for instance, employed magnetic deformation of a PDMS microfluidic device hosting primary fibroblasts and keratinocytes at a subdermally perfused air–liquid interface. The cyclic mechanotransduction caused wrinkles after one week and decreased the production of structurally important proteins [113]. By contrast, Mori et al. observed that cyclic stretch in their similar—but vascular‐perfused, with HUVEC‐lined needle‐templated channels—skin model yielded improved morphology (Figure 3a) [114]. After 4 days, they demonstrated a thicker outer skin layer, better resembling human skin, and a strengthened basement membrane and higher cell density and collagen production in the second layer. The discrepancy may be reminiscent of the different effects of mechanical skin stimuli observed at different time scales in vivo [115], though further study is clearly required.

The need for and potential utility of advanced multi‐layer OoC has been demonstrated beyond the discussed disorders also for edema or infection [121, 122]. Factors like skin state, age, and degree of hydration influencing skin penetration, reinforce the need for individual and adjustable in vitro models also for transdermal drug transport [3]. While not yet realized, all the ingredients are now available for iPSC‐derived, immune cell‐populated, vascularized, and innervated skin‐OoCs that recapitulate all major decisive factors for individual disease modeling and evaluation of drug responses.

### 3.4 Ocular Barriers

The ocular system comprises a wide range of barriers. The specialized epithelium of the outer blood–retinal barrier (BRB) provides the functional interface with the sensory cells. A specialized EC barrier, structurally and functionally analogous to the BBB (see Section 3.2.1; here termed the inner BRB), prevents neurotoxicity and controls the nutrient and metabolite transport to the neuronal tissue. The cornea, conversely, functions as the outermost partition of the eye against the outside world. In vitro research, and personalized OoC in particular, has to date exclusively focused on these three, which we will review in respective sections below.

A number of additional barriers are present, notably along the internal interfaces of the aqueous humor‐filled anterior cavity behind the cornea. This includes the lens, iris, and specialized epithelia and endothelia dealing with balanced aqueous humor turnover. These have received less attention, though simpler in vitro models have emerged. 3D primitive lenses, for instance, can be generated from iPSCs, with specific patient‐derived lines showing some promise to study genetically driven cataract (i.e., lens clouding) development [123]. Dysfunctional resistance and drainage of aqueous humor, meanwhile, are characteristic of glaucoma, where a pathological increase in intraocular pressure leads to progressive degeneration of retinal ganglion cells, and which has a number of known genetic risk variants [124]. Given that some 3D culture constructs (here, primary cell‐based, with a lack of iPSC‐based approaches) have better recapitulated physiological cell interaction, morphology, and stress response of the drainage structures, it suggests that these barriers may also become an insightful target for OoCs in the future [124, 125].

#### 3.4.1 Outer Blood–Retinal Barrier

The outer BRB is formed by a monolayer of tightly interlinked retinal pigment epithelium (RPE) overlying the microvascular bed of choroidal capillaries at the eye wall [126]. The cell populations are separated by Bruch's membrane, a specialized and at 4 μm, relatively thick ECM membrane consisting of multiple layers. Unique features of the RPE include its direct contact with neural tissue and its role in the regular phagocytosis of photoreceptor cells' outer segments. The choroidal capillaries can reach among the highest perfusion rates in the human body, nourishing the retina, the tissue with the highest oxygen consumption per unit weight [126, 127]. Further facilitating this supply function, the choroidal capillary ECs are fenestrated and thus quite permeable to nutrients and large macromolecules. Maintaining balanced choroidal neovascularization is essential for healthy retinal function [128].

Availability of human ophthalmic tissue and adult RPE is very limited [129]. Addressing the additional issue of dedifferentiation, Gu et al. were curiously able to avert this by adding supernatant from iPSC cultures to primary RPE [130]. Furthermore, they observed that RPE cultured on small scaffolds of laser‐cut cornea tissue exhibited more finger‐ and hair‐like structures (microvilli and cilia) critical for RPE functions, indicating the importance of microenvironmental control. At the same time, iPSC‐RPE with key structural and functional characteristics of healthy RPE—including cell polarity, effective phagocytosis, and organelle motility—have been generated from iPSCs, presenting an attractive basis for disorder modeling [131]. Protocols for differentiation of iPSC‐EC with enhanced expression of choroidal‐enriched genes have also since been published [132].

One relevant class of disorders is degeneration of the macula, that is, the retinal area with the highest photoreceptor density, resulting in loss of central vision. In addition to a number of specific genetic variants, even the more prevalent and partially environmental age‐related macular degeneration has a sizable inheritable component [128, 133]. To better understand the involvement of RPE, RPE‐secreted or serum factors, and choroidal ECs, Manian et al. developed a static, Transwell‐hosted 3D model, including iPSC‐RPE, generic iPSC‐ECs, and MSCs [134]. Their hydrogel‐embedded ECs formed a 3D choriocapillary‐like network—notably including fenestrations—a process that was shown to rely on factors secreted by both the RPE monolayer on top and the stromal monolayer underneath. Comparing cells from patients with a monogenic macular dystrophy to healthy controls revealed a decreased amount of a fenestration‐associated protein in the patient‐derived vascular networks. The researchers further showed that factors secreted from patient‐derived iPSC‐RPE, or exposure to patient serum, were sufficient to start choroidal neovascularization. Both of these phenomena are consistent with clinical findings, and the researchers confirmed inhibitory targeting of pro‐angiogenic factors as potential treatment options. Paek et al. translated this type of outer BRB model into an OoC by seeding iPSC‐RPEs on top of a self‐assembled vascularized hydrogel (Figure 3b) [116]. This capillary bed, from primary retinal ECs and choroidal fibroblasts in a collagen‐fibrin matrix, could be perfused via two adjacent needle‐templated, EC‐seeded channels inside the gel. Compared to iPSC‐RPE monoculture, the researchers noted enhanced basement membrane formation (reminiscent of Bruch's membrane) and pigmentation of the RPE. Arık et al. developed a slightly simpler outer BRB‐OoC, with cell line RPEs above a single needle‐templated vessel (HUVECs) [135], but subsequently showcased an isogenic iPSC‐derived version [136]. Although the results—including improved vascular integrity under co‐culture—are preliminary, these advances support the development of personalized platforms for macular disorders and therapeutics.

Other researchers have focused on recapitulating additional retinal complexity on chips. One group used iPSC‐derived retinal organoids, harboring the known essential retinal subtypes such as ganglion cells, bipolar cells, horizontal cells, and photoreceptors [137]. These organoids were embedded in a hyaluronic acid‐based hydrogel and deposited on top of iPSC‐RPE monolayers, with microfluidic perfusion underneath (separated by a porous membrane only). By maintaining the viability of retinal organoids during long‐term cultivation for over 1 year, this retina‐OoC could in the future enable close investigation of disease initiation and progression. Researchers from the same group later developed a complementary model focusing on features lacking earlier, that is, choroidal capillaries and immune interactions [138]. Their triple‐layer vertical/membrane OoC contained a bottom (stromal) compartment with melanocytes embedded in hydrogel, a perfused central channel fully lined with microvascular ECs, and a top channel hosting iPSC‐RPE. Cipriano et al. were thus able to recapitulate in vivo‐like choroidal structure and function, including a tighter barrier at the EC‐RPE interface and higher permeability at the EC–stroma interface. Perfusing with human peripheral blood mononuclear cells and activated T cells enabled analysis of immune cell migration and cytokine response, relevant to inflammatory conditions such as uveitis.

In the future, fine‐tuning the thickness and composition of Bruch's membrane in outer BRB models could enhance accuracy in simulating biologically relevant transport processes [127, 139]. Additionally, incorporating clinically relevant readout techniques, such as fluorescence tracing, will allow for precise comparisons with patient data, further supporting translational research and advancing treatment development [135].

#### 3.4.2 Inner Blood–Retinal Barrier

Compared to the outer BRB models discussed earlier, progress on the highly selective inner BRB (with its BBB‐analogous structure; cf. Section 3.2.1) has been lagging [140]. Early work with primary or immortalized retinal ECs by Ragelle et al. highlighted the importance of perfusion as well as matrix stiffness [127]. Employing a commercial lateral/hydrogel chip, they illustrated significantly lower barrier permeability compared to static culture or stiff plastic membrane supports. Maurissen et al. later developed a more comprehensive inner BRB‐OoC based on network self‐assembly of primary human retinal microvascular ECs in a fibrin gel co‐seeded with retinal PCs and retinal astrocytes [141]. Compared to EC monoculture, tri‐culture proved critical to the formation of reproducible and stable networks. Upon diabetic stimulation (glucose and cytokines), their model successfully recapitulated clinical features of non‐proliferative diabetic retinopathy, including PC death, vascular degradation, ECM dysregulation, and inflammation. This suggests that future studies with patient‐derived BRB cells in well‐engineered microenvironments should improve understanding of ocular diseases and potential treatments.

#### 3.4.3 Cornea

The anterior portion of the eye is formed by the cornea, which functions as a protective barrier against dust, dirt, and pathogens, while ensuring proper light refraction onto the retina for visual acuity. This specialized transparent tissue is composed of three primary cellular layers [142]. First, the constantly renewed outermost epithelium contains three sublayers with characteristic cell densities and morphologies. Limbal stem cells at the corneal periphery give rise to basal layer cells, which, in turn, differentiate into wing and superficial cells [142]. At the periphery, the corneal epithelium transitions into the conjunctival epithelium, a goblet cell‐containing stratified layer supported by underlying connective tissue. This structural continuity ensures a seamless protective barrier across the ocular surface and contributes to tear film stability [143]. Second, the thick stroma—accounting for approximately 90% of the total—consists of precisely arranged collagen fibers that provide structural integrity and transparency [142]. The stroma is maintained by resident corneal fibroblasts (keratocytes) and separated from the adjacent layers by a specialized basement membrane. Finally, a monolayer of hexagonal ECs regulates the fluid balance and thus preserves corneal hydration and clarity. The corneal endothelium exhibits a peripheral‐to‐central density gradient; its inability to regenerate leads to a gradual decline in cell density with age, impacting corneal function over time [142].

As highlighted earlier, the availability of human ophthalmic tissue for in vitro studies is restricted. Insights into early corneal development have guided strategies to differentiate corneal iPSC‐epithelial cells, ‐ECs, and ‐keratocytes—initially employing various ocular cell‐conditioned media, later using isolated signaling pathways [144]. The distinct germ layer origins of these cells have also prompted the development of a protocol for concentric “whole eye” co‐differentiation, giving rise to various regional progenitors, including surface ectoderm‐derived corneal epithelium suitable for use in corneal regeneration [145]. These strategies have already proven valuable in disease modeling, as seen, for instance, in iPSC‐keratocytes derived from keratoconus patients—a disease characterized by stromal thinning and curvature deformation—that show impaired survival and proliferation [146]. The known impact of curvature‐induced mechanical forces on disease‐implicated factors like cell migration, focal adhesion dynamics, and matrix remodeling is just one example of the need for advanced corneal OoCs [142].

Corneal blindness mainly arises from epithelial injuries after infection or mechanical trauma, and a number of genetic dystrophies affecting the different corneal layers have also been documented [142]. OoC models to date have chosen to focus on the more direct injuries, still illustrating complex stromal wound healing processes. Deng et al., for example, developed a cornea‐OoC to model bacterial keratitis, an inflammatory condition triggered by bacterial invasion of the corneal stroma following an injury [147]. Using immortalized human corneal epithelial cells cultured at the air‐interface on top of collagen‐embedded primary human corneal fibroblasts, they compared intact models to those with a reproducibly engineered epithelial defect under exposure to *Staphylococcus aureus*. Seo et al., on the other hand, developed a biomimetic ocular system incorporating blink‐induced mechanical forces to model dry eye pathology [143]. This condition, driven by excessive water loss from the ocular surface, leads to tear hyperosmolarity and inflammatory cytokine production, and may, in severe cases, cause corneal injury. The researchers' model consisted of a dome‐shaped 3D scaffold containing primary human keratocytes embedded in a hydrogel, with an underlying perfusion chamber. To replicate the natural concentric cell organization, 3D patterning of primary human corneal epithelial cells and immortalized conjunctival epithelial cells was applied on top of the keratocytes. Dynamic tear film regulation was mimicked using a hydrogel eyelid analog, supplied by artificial tear liquid through channels in the chip and cyclically across the corneal model to facilitate liquid film formation and control artificial tear flow. Reducing the blinking frequency within this system successfully simulated the aforementioned pathological features of dry eye disease [143].

Reconstructing the structure and function of the corneal epithelium and endothelium in vitro remains challenging. The two advanced models discussed above are still lacking ECs, and elements such as innervation and immune system interactions remain neglected [142]. Integrating these complex physiological and mechanical cues into patient‐derived corneal models will be essential for advancing our understanding of corneal diseases and for developing targeted, patient‐specific therapies.

### 3.5 Aural Barriers

Like the ocular system, the aural system contains a variety of barriers. Critical for normal hearing is the blood–labyrinth barrier (BLB), a specialized capillary network that acts as a highly regulated, homeostasis‐maintaining barrier between the blood and the inner ear fluids (i.e., perilymph and endolymph) [148]. Similar to the BBB, the BLB consists of a tightly interlinked EC monolayer, surrounded and supported by PCs. Additionally, the BLB features perivascular‐resident macrophage‐like melanocytes that play an important role in maintaining barrier integrity [148]. In the cochlea, the BLB function is supported by the specialized epithelial cells of the stria vascularis, including marginal cells oriented toward—and helping maintain the unique ionic environment of—the endolymph [149]. The cochlea is further home to a complex epithelial structure composed of mechanosensitive sensory hair cells; these reside, along with a variety of support cells, on the specialized layered ECM of the basilar membrane [150, 151]. On the opposing side of the cochlea, the simpler epithelial vestibular membrane provides a barrier against the perilymph‐filled compartments (which ultimately connect with CSF) [152].

There are few surgical procedures in which healthy tissue of the inner ear would be obtained [148]. At the same time, we did not find attempts to differentiate BLB‐specific cells from iPSCs, though progress has been made in generating other inner ear cell types. Early on, human cochlear neurons and hair cells have been differentiated from fetal auditory stem cells [153]. More recently, iPSC‐derived inner ear organoids have shown promise by producing functional hair cells that display electrophysiological properties akin to native sensory epithelium [154]. Modeling the cochlea and its barriers holds relevance for disorders ranging from vascular malformation to autoimmune diseases that may result in vertigo, tinnitus, and progressive hearing loss [148, 155]. Some, like Meniere's disease—where a wide range of changes in the stria vascularis and basilar membrane have been documented—remain poorly understood but are influenced by genetics and epigenetics on top of environmental factors [152].

Still, progress on advanced cochlear models also remains limited. Sekulic et al. only recently developed the first BLB‐OoC using post‐mortem‐derived ECs and PCs [148]. The vertical/membrane‐style chip was built out of medical‐grade adhesive tape, a simple and low‐cost approach incidentally building on our own prior work [156]. Cell growth as well as barrier integrity and size‐selective drug permeability were validated within the perfused OoC. However, the authors did not evaluate functional characteristics against static conditions. For sensory epithelium, we were unable to identify even a single human OoC. Hu et al. did demonstrate how OoC‐integrated electrical stimulation (controlled, fittingly, by a cochlear implant) could enhance sensory epithelial differentiation when applied across *murine* cochlear organoids in their microfluidic culture chamber [157].

Reproducing the complex architecture of the inner ear in vitro thus remains a challenge. Recent advances in manufacturing technologies, including 3D bioprinting, and in iPSC‐based protocol development hold great potential for generating physiologically relevant OoCs [158]. Such systems could significantly improve our understanding of how interactions between the different barrier systems contribute to inner ear disorders and could thereby enable the development of new therapeutic strategies.

### 3.6 Respiratory System

For our purposes, the respiratory system can be divided into the airways (providing passage for ambient air) and the lungs (mediating oxygen exchange). These compartments feature distinct structures and cell ensembles, and we will review them separately below. As the primary bodily interface with air, the respiratory system and its modeling are naturally of interest to viral infectious diseases like influenza or COVID‐19; it is also the organ with the highest cancer mortality. Regarding fully personalized models, however, cystic fibrosis (CF) is the most relevant [159, 160]. CF is one of the most prevalent genetic disorders and simultaneously has a high degree of genetic variability in the specific defect on the CFTR gene [161]. It inhibits mucus clearance and can thus lead to death from respiratory complications (it also affects the GI tract, see Section 3.7). The spectrum also extends beyond to primary ciliary dyskinesia (also a clearance disorder) and the genetic factors involved in asthma or chronic obstructive pulmonary disease, such as the even more prevalent α1‐antitrypsin deficiency [162].

#### 3.6.1 Airway

In the airway, the epithelium consists primarily of basal, secretory, and ciliated cells, with basal cells being stem cells of the latter two cell types [163]. Together these cells produce mucus that traps particles, then transport this mucus back out of the airways [6]. Brush cells and pulmonary neuroendocrine cells also occur in the airway epithelium but are rare. The exact structure and composition of the airway epithelium varies depending on location (proximal vs. distal). The olfactory epithelium is particularly specialized, with its olfactory innervation and glial‐analogous support cells [164]. Throughout the airways, balanced presence and actions of antimicrobial proteins, the resident microbiome, epithelial‐derived cytokines, and various immune cells are essential [165, 166].

Primary upper airway epithelial cells are relatively easy to obtain and maintain; their accessible location allows collection via a simple brush swab, and cultured cells exhibit high proliferative capacity [167]. Probably because of this high availability of primary cells, no efforts have been made so far to differentiate iPSCs into upper airway epithelial cells. Olfactory epithelium stem cells that continuously enable tissue renewal serve as a valuable source for in vitro cultures, based on which nasal and olfactory epithelium organoids could be established. Patient‐derived organoids have been employed to study, for instance, chronic rhinosinusitis, a common inflammatory disease affecting the nasal mucosa and paranasal sinuses [164]. By contrast, at least one iPSC organoid‐based protocol has already been developed to derive lower airway progenitors that show diversification into cells with hallmarks of the three major epithelial types [168]. The use of these organoids in personalized disease modeling has been demonstrated before, as organoids derived from CF patient iPSCs exhibited specific loss of CFTR function and individualized responses to therapy [169].

The physical environment of the airways is primarily characterized by bi‐directional airflow. Park et al. recently demonstrated that this contributes to physiological function even with a simple lung‐derived cell line, observing improved goblet cell differentiation and a more pronounced glycocalyx compared to a static air–liquid interface in their OoC [170]. The biochemical environment naturally remains an important factor, with cocultures of primary nasal epithelial cells and ECs on opposite sides of a lateral/hydrogel interface revealing that EC signals promote mucosal maturation and nasal gland‐like structures even at a static air‐interface [6]. Walls et al., conversely, studied primary human nasal epithelium lacking EC support under physiological airflow [171]. Interestingly, while 24‐h airflow exposure upregulated the mucus production, they demonstrated that pre‐conditioning their vertical/membrane OoC with sub‐physiological airflow already during the differentiation phase eliminated this. Given also concomitant reduced cytokine secretion, they consider this mucus response an inflammatory hypersecretion to an otherwise‐sudden change in stimulation, indicating the need for multi‐modal assessment. Shrestha et al., in fact, employed short‐term (<1 h) pressure‐driven airflow exposure to model continuous positive airway pressure (CPAP) on the upper airways [172]. CPAP is the most effective treatment for sleep apnea, a chronic disease characterized by reduced or stopped airflow. More pronounced even than the findings of Walls et al., they document upregulated markers of inflammation and cell stress in their nasal epithelial cell line‐based Transwell‐style OoC. The researchers link this to known CPAP side effects, but their lack of independent pressure/flow regulation and their choice of control as zero pressure/flow (i.e., resembling sleep apnea), present limitations.

Toward modeling airway fibrosis, Mejias et al. designed a six‐channel platform in two layers [173]. The vascular layer consisted of a central chamber with a self‐organized network of HUVEC endothelium in a gel with the fibroblasts of interest. Flanking channels in the same layer provided nutrients from the medium, as well as cytokine gradients from healthy primary fibroblasts. In the second layer, the airway channel—hosting primary small airway epithelium—was positioned on top of the central vascular channel, separated by a collagen‐cast membrane. For simplified handling, 8 such devices were fit into a standard 96‐well plate format. The researchers proceeded to model two diseases. For idiopathic fibrosis, they compared healthy against patient‐derived fibroblasts in the central vascular chamber, and were able to observe induction of more fibrotic phenotypes in the (intrinsically healthy) epithelium. For CF, they instead compared healthy versus patient‐derived epithelial cells in terms of neutrophil migration, and showed increased recruitment of primary neutrophils into the diseased airway‐adjacent vasculature. This model did lack biomechanic stimulation, however, and did not incorporate specialized respiratory capillary ECs. Plebani et al. fully relied on primary cells for their model, culturing healthy or CF bronchial epithelial cells and healthy lung microvascular endothelial cells on opposing sides in a commercial vertical/membrane chip [174]. Similarly to the work by Mejias et al., this model also showed increased neutrophil recruitment, and upon infection with *P. aeruginosa* bacteria, the mucus of the CF‐derived OoCs contained more bacteria compared to healthy controls. In addition, the bacteria grew faster in mucus extracted from the perfused CF devices compared to mucus from a static Transwell system.

Future personalized applications could also include asthma and nasal allergies, where diminished tight junctions were documented, indicating the relevance of epithelial dysfunction for the initiation or progression of allergic diseases [175, 176]. Nawroth et al. developed an airway OoC to study rhinovirus‐induced exacerbation of asthma symptoms, though they utilized primary cells from healthy donors, using IL‐13 stimulation to mimic allergic asthma [177]. Further integrating components of the airway microbiome and immune system could be crucial, as it is assumed, for instance, that T cells could react to mucosal germs with a direct induction of a type‐2 mucosal inflammation [178]. A holistic understanding of respiratory disease mechanisms may also require studying interconnected systems of airways and lungs—discussed in more detail below—as suggested by Nof et al. [179] Interconnecting three OoCs with distinct fluidic geometries and cell line populations, this enabled them to simulate airflow and viral spread from nasal passages to alveoli.

#### 3.6.2 Lung

The alveolar–capillary barrier is the main functional unit of the lungs, facilitating oxygen and carbon dioxide exchange between the blood and air inside the lungs. The barrier consists predominantly of alveolar epithelial cells, which are in close interaction with underlying vascular ECs to facilitate blood oxygenation [180]. The epithelium is mainly made up of two subtypes, termed AT1 and AT2. AT1 cells are terminally differentiated, flat, squamous cells that provide the bulk of the gas exchange surface in the alveolus. AT2 cells, conversely, are cuboid cells that secrete the surfactants of the air‐facing alveolar lining layer and that act as epithelial stem cells, being capable of self‐renewal and differentiation into AT1 cells [163]. Other more rare cell types in the epithelium are brush cells and pulmonary neuroendocrine cells, each making up less than 1% of the alveolar surface, playing a role in the detection of bacterial infections and hypoxia, respectively.

Patient‐derived primary cells for lung modeling require more invasive (compared to upper airways) lung biopsies or surgical resections [159]. Still, the self‐renewing capabilities of resident stem cells make them an attractive alternative. Early iPSC differentiation attempts involved culturing embryonic stem cells in media optimized for primary alveolar cell culture with only limited efficiency [181]. More efficient methods since then have chemically followed the various steps of in vivo development, from iPSCs to definitive and ventral anterior foregut endoderm to lung progenitor cells to AT2 cells [182, 183, 184]. Once AT2 cells have been obtained, they can be differentiated further toward AT1 [185].

The term “OoC” was first published with a lung OoC [186], and the very first microfluidic mammalian culture model also included a lung cell compartment [187]. OoCs are attractive platforms for lung models, as they can allow for cyclical breathing‐like strain, in addition to shear stress at the vascular side by fluid flow and the epithelial side by airflow [188]. The “standard” lung OoC features a porous cell culture membrane from stretchable PDMS, with alveolar epithelium and ECs on opposing sides, and the corresponding channels, respectively, exposed to air and perfused by media. Pneumatic actuation of the device enables cyclic stretching to recapitulate breathing. With an alveolar epithelial cell line and primary alveolar ECs, Huh et al. originally demonstrated more in vivo‐like nanotoxicological modeling compared to static cultures [186]. Stucki et al. later showed superior physiological lung function in a similar but fully primary cell OoC over 3 weeks, with additional technological innovations like thinner membranes and higher parallelization [189, 190].

Personalized disease lung‐OoC applications nevertheless remain rare. In an effort to model pulmonary thrombosis, Jain et al. adapted the original OoC of Huh et al. to feature primary pulmonary epithelial cells, as well as vascular perfusion with whole blood [191]. Pulmonary exposure to bacterial endotoxin (LPS), known to induce thrombosis in mice [192], led to the formation of thrombosis in the chip. The model's potential for drug screening was demonstrated by introducing an experimental modulator of tissue inflammation and hemostasis, which led to a decrease in thrombi formation and barrier leakage (similar to in vivo results, albeit from mice). Recently, Gagnon et al. incorporated iPSC‐AT2 cells in an alveolus‐OoC (Figure 3c) [117]. The epithelium was seeded in a blunt‐ended channel adjacent to a HUVEC‐seeded perfusable channel, both with cylindrical lumens inside collagen I. They demonstrate that the iPSC‐AT2 cells form a monolayer while maintaining AT2 characteristics. Furthermore, by using iPSC‐AT2 cells with the KRAS G12D oncogenic mutation, they were able to show changes in epithelial morphology and an increase in angiogenic sprouting in the vascular channel, a result of the paracrine communication between the compartments. Lastly, Amoakon et al. sought to investigate the effect of CF on the lung microvasculature and cultured primary CF ECs under shear stress in a microfluidic system [193]. Where the bulk of research into CF focuses on the epithelial effects (cf. Section 3.6.1), their work shows that CF also affects endothelial homeostasis by modulating EC mechanosensitive channels, leading to barrier breakdown, impaired angiogenesis, and loss of small pulmonary vessels.

Leveraging the technological capabilities of lung‐OoCs has already advanced our understanding of lung physiology and biomechanics, though such OoCs' application toward disease modeling has, in comparison, been more limited. While more recent efforts have incorporated iPSC‐derived cells into lung‐OoCs to study diseases such as lung cancer and cystic fibrosis, personalized applications remain in the early stages. Continued refinement of differentiation protocols and chip design is therefore essential to fully realize the promise of these platforms in personalized disease modeling and drug discovery.

### 3.7 Gastrointestinal System

The GI system is another primary contact surface of the human body with the outside world, focused primarily on nutritional uptake. We consider it in four parts: First, the oral mucosa, which naturally also plays a role in respiration and shares similarities with the nasal/airway mucosa discussed in Section 3.6.1, alongside the esophageal mucosa. Second, the gastric and intestinal mucosa, ranging from stomach to colon. Finally, we will consider the many accessory organs of digestion, divided into the biliary tract and the pancreas.

#### 3.7.1 Oral and Esophageal Mucosa

As a primary gateway for food compounds, airborne particles, and microbes, knowledge about the barrier properties of the oral mucosa is important for infections, cancer, and drug delivery, and holds unique relevance for dentistry [194]. Most of the oral cavity is covered with lining mucosa, a multilayered stratified squamous epithelium, which also characterizes the esophagus [195, 196]. In masticatory mucosa, an outer layer of keratinocytes helps endure constant mechanical stress or injuries. Finally, certain areas of the tongue are covered with highly specialized mucosa that combines supporting and gustatory cells (not themselves neurons, but basally coupled to sensory neurons) [194]. Different stem cell types, namely mesenchymal, adult, and tissue stem cells, are present, including also the tissue enclosing the developing tooth germ or the dental pulp mesenchyme [197]. Compared to cutaneous wounds, oral mucosal injuries recover more quickly with less inflammation, which could be explained by increased migratory and proliferative capabilities of oral keratinocytes and the range of stem and immune cells located in the oral mucosa [198, 199]. As in the airways, a diverse microbiome is crucial for mucosal homeostasis and kept carefully balanced by secretions of antimicrobial proteins and cytokines, along with shedding and a high turnover [199, 200].

Also, as with the upper airways, accessing primary oral tissue is relatively simple, and a plethora of non‐cancerous immortalized keratinocytes have been derived from various oral mucosa compartments [194]. Perhaps it is due to this ease and abundance of relevant cell types that differentiating oral cells from iPSCs for generation of individualized oral tissue models has not yet been pursued [199]. Salivary gland spheroids and organoids, which can be derived from primary cells, have proven valuable for studying salivary gland pathophysiology [201]. Song et al. developed a method for isolation of intact human acinar cell clusters and intercalated ducts that retain key properties of intact glands, including secretory function and both cell–matrix and cell–cell interactions [202]. To advance research on head and neck cancers, chip‐based models have been designed to replicate the complex environment of soft, glandular, and hard tissues in this region [203]. As it is more difficult to obtain primary human esophageal cells, iPSC‐based models have been developed and have shown promise in modeling congenital developmental anomalies. Raad et al., for instance, differentiated iPSCs from afflicted patients into esophageal epithelial cells and organoids via an endoderm and anterior foregut stage [204]. The researchers identified distinct transcription patterns and isoforms for patient‐derived cells, particularly in the developmental stage, and were able to show that closely related tracheal development is unaffected (matching clinical observations).

An oral mucosa‐OoC was developed by Rahimi et al., containing three microchannels separated by micropillars. Human gingival immortalized fibroblasts were embedded in collagen in the middle channel, while keratinocytes were located in the pillar gaps [200]. This OoC enabled layer‐specific responses to bacterial and toxic stimuli occurring in the oral environment and showed greater sensitivity in toxicity screening than monocultures [5, 200]. The researchers subsequently incorporated also dermal ECs to study cancer therapy‐induced oral mucositis and extended culture viability by stabilizing the collagen matrix [205].

Barrett's esophagus is a condition in which the lining of the esophagus changes due to chronic gastroesophageal reflux and could sometimes lead to the development of esophageal adenocarcinoma. Shimshoni et al. used an esophagus‐OoC to study the role of stromal‐derived fibroblasts in this epithelial transformation and cancer development [206]. Primary fibroblasts were isolated from either healthy esophageal tissue or from (a)symptomatic regions of patients' esophagi. These cells were embedded inside a collagen gel (with media perfusion underneath), and patient‐derived esophageal epithelial cells were seeded at the upper air–liquid interface. When co‐cultured with healthy fibroblasts, the epithelial cells formed flat, multi‐layered structures resembling normal esophageal tissue. By contrast, co‐culture with patient‐derived fibroblasts resulted in epithelial layers that exhibited cyst‐like or closed glandular structures and expressed a mix of gastric and intestinal mucins—features that reflect the variability of tissue changes observed in patients. Pal et al. subsequently built on this approach in a commercial vertical/membrane chip toward predicting chemotherapy responses [207]. Treatment‐naive patient tumors or adjacent normal tissues were isolated and cultivated as organoids, then fragmented and seeded into the upper channel of the chip. Patient‐matched regional cancer‐associated or normal fibroblasts were seeded into the stromal channel below. This patient‐specific stroma‐inclusive esophagus OoC demonstrated predictive value of chemotherapy outcomes within approximately 12 days, a time frame considered clinically relevant.

To date, genetic backgrounds beyond cancer have been largely neglected in mucosal models. Complex patient‐derived models—including immune cells and mouth bacteria—could resolve unknown factors for the onset and progression of periodontitis and related diseases that are largely influenced by genetic susceptibility [195, 200]. Replacing or supplementing cell culture media with exogenous or locally secreted saliva, and incorporating active apical perfusion, was also thought to establish a more natural environment [199].

#### 3.7.2 Stomach and Intestine

The GI tract exhibits varied regional specializations in structure and function, reflecting its roles in digestion, absorption, and barrier maintenance. Resident stem cell populations throughout the GI tract enable continuous epithelial renewal, ensuring tissue integrity and function despite exposure to mechanical stress and environmental insults. Turnover rates vary by region: whereas the intestinal epithelium is one of the fastest (weekly)‐renewing tissues in the human body, the colon has a slower turnover, and the stomach epithelium regenerates at an even slower rate [208, 209]. A characteristic 3D crypt‐villus architecture is formed by periodic folding of the inner wall of the small and large intestine. The polarized epithelial monolayer lining these walls contains, besides the absorptive enterocytes, also mucin‐producing secretory goblet cells [210]. A small population of hormone‐secreting enteroendocrine cells help regulate intestinal motility, metabolism, and mucosal immunity [210]. The deepest regions of the tissue folds, the crypts, host the stem cell niche alongside specialized secretory cells producing bactericidal substances like lysozyme that support it [208, 210]. The colon hosts similar cell populations with similar, though regionally adapted function, but notably lacks villi [211]. In the stomach, specialized glandular units within the gastric epithelium secrete digestive enzymes, acid, hormones, and mucus. Its epithelium is adapted to withstand the particularly harsh acidic environment while supporting digestion.

The GI epithelial barrier is constantly exposed to external agents and antigens, such as nutrients, toxins, or pathogens. Moreover, orally administered drugs are mainly absorbed at the small intestine, which is also largely involved in drug metabolism, expressing both drug transporters and drug‐metabolizing enzymes such as cytochrome P450 [212]. Intestinal homeostasis is preserved by diverse host–microbiome interplay and immune reactions across the intestinal mucosa [118]. These complex reciprocal actions, involving also the neighboring endothelial tissue, are dynamic and context‐dependent [210]. For instance, chronic relapsing inflammatory GI states, known as inflammatory bowel disease (IBD), comprise ulcerative colitis (UC) and Crohn's disease (CD) and seem to be triggered by microbiota, environmental factors, or diet in genetically susceptible individuals [213]. Like IBD, celiac disease results from the interplay of genetic susceptibility and environmental triggers, with gluten exposure initiating autoimmune damage in the small intestine [214]. In all cases, susceptibility might be conveyed by mutations affecting the integrity of intestinal epithelium and immune regulation [213].

Primary cells are somewhat widely available for GI models due to the prevalence of biopsies after endoscopic or surgical interventions. Diverse intestinal cell types can be obtained also with specificity for different gut regions [215, 216]. Still, Takayama et al. attempted to generate human intestinal iPSC‐epithelial cell monolayers, and demonstrated drug absorption rates and expression levels of a cytochrome P450 subtype correlating well with the human small intestine in vivo [212]. More complex intestinal and also gastric organoid cultures have been derived from both primary stem cells or iPSCs, and accomplished, for example, intestinal villi formation and mucus production, or gastric domain‐specific secretion profiles [210, 217]. For studying GI barrier function, challenges arise, however, with regard to accessing the enclosed lumen (typically apical‐in; reversed‐polarity protocols have since been developed) [215, 218]. Alternatively, GI organoids can be “opened” by enzymatic digestion and re‐seeded (after optional cell selection/sorting) onto Transwells or into OoC [118, 213]. Besides tissue‐tissue intercommunication with adjoining endothelium and circulating immune cells, GI OoC can recapitulate in particular the complex biomechanics from peristalsis [118, 219].

One of the few extant stomach‐OoC, supporting robust 3D growth of human iPSC‐derived gastric organoids, was presented by Lee et al. [220]. A single organoid, embedded in Matrigel, was placed into a central chamber, and borosilicate micropipettes were inserted into opposing sides of the organoid. Connecting a peristaltic pump allowed the researchers to establish not only luminal flow through the organoid, but also cyclic expansion matching the pumping cycle. The cannulation of organoids opened potential of luminal delivery, beneficial for both long‐term supply with nutrients and for analyses of gastric diseases and screening of oral drugs. By contrast, Hofer et al. utilized dissociated organoids (derived from healthy gastric biopsies) in an OoC for bilateral access [221]. Instead of a membrane, they employed a stamp‐formed collagen I hydrogel mimicking gastric epithelial morphology as the culture support, with perfusion capabilities above and below. Under apical acidic (i.e., physiological) conditions, the OoC promoted enhanced maturation of glandular‐resident pit cells compared to conventional organoid cultures. They were further able to study the common gastric infection route of *H. pylori*, including niche formation, colonization, and an antimicrobial response of pit cells previously observed in mice but not human cells. At the other end of the GI tract, colon OoCs are similarly not much present in the literature. Sontheimer‐Phelps et al. developed one, relying on fragmented primary organoids inside a commercial vertical/membrane chip [216]. Under perfusion culture (though without stretch), they observed generation of a mucus layer with thickness and bilayered microstructure characteristic of the human colon. Perfusing Prostaglandin E2—an endogenous mediator involved in UC—through the basal channel showed increased cell proliferation and mucus swelling, in line with some of its documented in vivo effects.

Research on intestinal OoC has been much more extensive. Naumovska et al. demonstrated the direct differentiation of iPSCs into intestinal‐like tubules within a commercial lateral/hydrogel OoC system [222]. These tubules resembled the functional adult intestinal epithelial barrier with apical–basal polarity, expression of transporters, and higher sensitivity to inflammatory cytokines than cell line‐derived models. Beaurivage et al. used the same OoC platform to culture dissociated intestinal organoids derived from healthy patient biopsies, but also incorporated primary macrophages to mimic immune‐epithelial interactions [213]. By exposing the cells to interferon‐γ and LPS, they were able to model certain aspects of inflammatory bowel disease, such as M1 macrophage polarization and epithelial cytokine release. More recently, Moerkens et al. focused on further improving the physiological relevance of intestine‐OoCs by developing a model with a more complex, in vivo‐like architecture [223]. Building on earlier work by, for example, Workman et al. [224], epithelial cells were selected from iPSC‐derived intestinal organoids and cultured in the top channel of a commercial vertical/membrane chip. To mimic growth factor gradients observed in vivo, cells were exposed to expansion and differentiation medium. This resulted in the generation of villus‐like structures in the top channels and a supportive subepithelial layer in the bottom channel. Interferon cytokine stimulation activated disease‐relevant pathways—mirroring clinical datasets—with the response mainly driven by the subepithelial tissue. These findings highlight how stem cell‐derived gut‐OoCs are evolving toward more physiologically accurate and patient‐relevant models for studying inflammatory disorders such as IBD and irritable bowel syndrome.

Yet most OoCs to date have stopped short of using disorder‐specific cells. Using primary organoids obtained from UC or CD patient biopsies, differentiation of disease‐specific 3D epithelial layers was first achieved under physiodynamic conditions by Shin et al. (Figure 3d) [118]. In a variation on the standard vertical/membrane chip design, the authors employed serpentine microchannels (rather than straight). The resulting multiaxial barrier deformations from vacuum actuation as well as asymmetric flow profiles allowed for a closer imitation of the dynamic intestinal biomechanics. While epithelial 3D morphologies were similar between healthy and both IBD conditions, the mucus layer was compromised for UC‐derived intestinal models. This is in line with lower mucin expression in UC patients. The authors were also able to show more significant structural changes with colorectal cancer‐derived epithelia, as well as a proof‐of‐concept anoxic–oxic interface to facilitate co‐culture with fecal microbiome samples; the major limitation for all results here being reliance on only a single donor in each condition. Similarly, Kharaghani et al. used primary human intestinal epithelial organoids from both healthy individuals and CD patients [225]. They employed a more typical commercial vertical/membrane chip, but they did expand on aforementioned works by including also primary intestinal ECs (healthy, non‐isogenic). Their study focused on micro‐ and nanoplastics, which did not impact healthy controls, but in the CD‐derived OoC somewhat surprisingly dysregulated a small number of genes in spite of reduced epithelial uptake. Building on the need to better understand the molecular, (epi)genetic, and nutritional bases of diseases, Bein et al. pursued a similar OoC approach to investigate environmental enteric dysfunction [226]. The condition is characterized by chronic inflammation of the small intestinal lining, structural changes in the gut, and reduced nutrient absorption. Following directly in the footsteps of Workman et al., they cultured dissociated primary organoids from patients or healthy controls in commercial vertical/membrane chips. When applying disease‐mimicking nutrient‐deficient medium, known reactions like villus blunting and barrier dysfunction were visible regardless of the cells' genetic background. Importantly, comparing the OoC with patient cells and healthy controls allowed them to distinguish cytokine responses and transcriptomic signatures typical for the disease and differentiate this from the externally‐induced scenario.

Taken together, specific models of the digestive tract could clarify initiation and progression of complex diseases involving multiple genetic and environmental risk factors [227]. Although progress has been made in modeling IBD and environmental enteric dysfunction, future patient‐derived OoCs for conditions like celiac disease may further support diagnosis of high‐risk individuals, and enable personalized treatment approaches [227]. Integration of genetic and epigenetic backgrounds with key features of the gut microenvironment, including immune responses and host–microbiome crosstalk, might elucidate disease etiologies and pathophysiological dynamics of disease developments in personalized contexts of diverse GI conditions [224]

#### 3.7.3 Biliary Tract

The biliary tract, an architecturally complex system, comprises the liver, bile ducts, and the gallbladder [228]. The liver conducts fundamental reactions as an exocrine and endocrine gland, including hormone and bile synthesis, and plasma protein secretion [229]. Bile ducts form a network of intra‐ and extrahepatic channels that facilitate the directional flow of bile, and the gallbladder stores bile until it is needed for digestion. Since the vast majority of ingested drugs are metabolized by the liver, evaluating hepatotoxicity is a critical component of early drug development [230]. Hepatic diseases, which are increasing in both prevalence and treatment cost, remain among the leading causes of death worldwide [229].

The blood–bile barrier ensures the division between blood in the intrahepatic microcirculation, known as hepatic sinusoids, and bile in the biliary canaliculi [231]. Hepatocytes, which perform vital liver functions such as synthesis, metabolism, and detoxification, also contribute to the blood–bile barrier through tight junctions that seal the bile canaliculi from the blood‐filled sinusoids. On the sinusoidal side, hepatocytes are adjacent to a distinctive fenestrated EC barrier that lacks structured basement membrane. This facilitates a fast molecular metabolic exchange between the bloodstream and hepatocytes, while still protecting hepatocytes from blood shear stress [230, 232]. Hepatic stellate cells reside in the space between sinusoidal ECs and hepatocytes, where they store vitamin A in their lipid droplets and secrete ECM components in response to liver injury. Kupffer cells, liver‐resident macrophages, are located within the sinusoidal lumen where they play a key role in immune defense and the maintenance of immune tolerance. The bile canalicular network is uniquely confined between the lateral membranes of adjacent hepatocytes, sealed by tight junctions that prevent bile leakage into the surrounding space. Hepatocytes actively secrete bile into these canaliculi, from which it flows into the bile ducts, and eventually goes to the gallbladder for storage, or into the intestine for digestion. During meals, the gallbladder is induced to rhythmic contraction and thus to expel bile into the duodenum [233]. The gallbladder and bile ducts are lined by tightly connected epithelial cells called cholangiocytes that play a central role in bile secretion, concentration, and reabsorption, thereby determining bile composition. Along the biliary tree, cholangiocyte morphology and function vary: in larger ducts and the gallbladder, they are predominantly large columnar cells, whereas in smaller ducts, they appear as cuboidal cells with the ability to proliferate or to differentiate into columnar cells during injury repair [233].

Primary human hepatocytes, liver sinusoidal ECs, stellate cells, and Kupffer cells can be isolated from resected liver tissue [234]. Co‐cultures of these major hepatic cell types were, for example, used to model steatotic liver disease and related inflammation and liver damage [235]. Cholangiocytes are additionally available from minimally invasive liquid (bile) biopsies [236, 237], as well as via hepatic progenitors or hepatocyte‐to‐biliary transdifferentiations [238]. While biopsies are relatively common, and the organs have relatively high regenerative potential, iPSCs nonetheless present a welcome alternative. Among iPSC‐cholangiocyte protocols, we highlight Sampaziotis et al., who developed organoids that replicated physiological hormone response, enzymatic activity, and bile acid transport. Using patient iPSCs, they succeeded in modeling three distinct genetic liver diseases as well as associated pharmaceutical interventions [239]. Many protocols have demonstrated that iPSC‐ECs and iPSC‐stellate cells can be generated via mesoderm induction, and that hepatic cues applied to macrophage precursors can lead to differentiation into iPSC‐Kupffer cells [240]. Most iPSC‐hepatocytes generated to date resemble primary hepatocytes functionally in, for example, plasma protein secretion and lipid storage, but show a deficit of cytochrome P450 activity [240]. Recent studies have shown that this limitation can be partially overcome through induction of certain transcription factors or multicellular organoid assembly [241, 242]. Further demonstrating the importance of an appropriate microenvironment, perfusion culture was shown to enhance aforementioned hepatic maturation and function both when applied to the on‐chip differentiation of iPSCs into liver organoids [243], as well as for culturing subsequently dissociated organoids in a commercial vertical/membrane chip (compared to intact ones in static culture) [244].

Steatotic liver disease is characterized by intracellular lipid accumulation in hepatocytes. The disease is mainly metabolic‐associated or alcohol‐associated, but genetic predisposition also plays a big role in the onset and progression of the disease [245]. Slaughter et al. approached metabolic dysfunction‐associated steatotic liver disease (and its ties with obesity and diabetes) with healthy primary hepatocytes and adipocytes, placed in separate chambers along the same microfluidic channel [246]. Under induced inflammation to mimic the disorder, the exchange of cytokines and adipokines within their OoC allowed them to observe adipocyte lipolysis and insulin resistance and confirmed the role of adipocytes in disease progression. Similarly, by applying clinically relevant blood‐alcohol concentrations to a tri‐culture of (again, healthy) primary hepatocytes, sinusoidal ECs, and Kupffer cells in a commercial vertical/membrane chip, Nawroth et al. sought to recreate key early characteristics of alcohol‐associated liver disease [245]. This liver‐OoC notably included the architecture of both liver sinusoids and bile canaliculi, achieved by sandwiching hepatocytes between two layers of optimized ECM to provide the necessary symmetric cell adhesion for correct polarization. Ethanol‐induced lipid accumulation and oxidative stress were exacerbated by simultaneous LPS exposure, highlighting the importance of a second insult in the disease progression. The researchers also observed altered expression of bile‐related genes and degradation of their biomimetic network of bile canaliculi, underlining these essential markers for alcohol‐associated liver disease.

Another chronic liver disease, even more closely linked to biliary malfunction, is sclerosing cholangitis (i.e., long‐term liver disease characterized by bile duct inflammation and scarring inside and outside the liver). To investigate the involvement of the vascular–biliary–stromal interface, Du et al. established a corresponding OoC [237]. They created two parallel, needle‐templated channels in a collagen matrix mixed with primary (healthy) gallbladder fibroblasts, and subsequently seeded HUVECs and cholangiocytes, respectively. The researchers found that their primary organoid‐expanded cholangiocytes (whether from surgical tissue or more readily accessible bile samples) organized into a compact epithelial monolayer. It exhibited functional barrier properties and transport activity for bile salt, independent of shear stress (which, in the vascular barrier, yielded reduced permeability). Comparing bile ducts from healthy subjects or patients with primary sclerosing cholangitis showed that, although many functional characteristics were conserved, there were changes in cytokine expression patterns when exposed to IL‐17A (previously implicated in the disease's signaling cascade). The observed stimulated inflammatory responses, and demonstration of correlated recruitment of vascularly‐perfused T helper 17 lymphocytes, indicate potential of the bile duct‐OoC for mechanistic analyses of relevant pathologies.

These examples underscore how OoCs incorporating biomechanical signals, a multicellular architecture, and patient‐derived cells create opportunities for investigating complex pathologies across the biliary tract. Furthermore, the use of iPSC‐hepatic and iPSC‐biliary cells offers valuable alternatives to primary cells, especially when modeling genetically caused diseases. Combining iPSC‐derived cells with advanced OoC platforms could greatly contribute to the development of precise, personalized liver disease models and the improvement of preclinical assessment of drug safety and efficacy.

#### 3.7.4 Pancreas

The pancreas is a structurally complex, multifunctional organ composed of diverse cell types organized into closely connected exocrine and endocrine compartments. These compartments secrete digestive enzymes and hormones, enabling the pancreas to respond to changes in blood glucose levels [247]. Whereas the endocrine signaling of the islets of Langerhans relies on a non‐specialized capillary EC network, epithelial ductal cells form a crucial mucosal barrier network [248]. This is responsible for delivering digestive enzymes from acinar cells to the duodenum and minimizing the risk of intrapancreatic digestive enzyme activation and retrograde flow [249].

Primary pancreatic cells can be obtained when a whole pancreas is removed in the context of inflammation [250]. Access to healthy pancreatic tissue is limited. Therefore, differentiation of pancreatic endoderm‐ or endocrine‐like cells from iPSCs and their utilization for research on diabetes mellitus was pursued by several research groups [247]. iPSCs have also been differentiated into pancreatic duct‐like organoids. Wiedenmann et al., for example, demonstrated this using custom microwells [248]. Time‐resolved single‐cell transcriptomics revealed that different routes from pancreatic progenitors through ductal intermediates generated distinct subpopulations of mature duct‐like cells, expressing mucins or also CFTR (cf. Section 3.6).

Diabetes is a frequent and severe complication of CF. Mun et al. hypothesized that this might originate from disrupted signaling between ductal cells and the insulin‐generating islet cells, taking into account their close proximity in the pancreas [250]. To test their hypothesis, the authors isolated respective cells from patients' surgically removed pancreas and co‐cultured them on opposing sides of a vertical/membrane OoC. It was observed that insulin secretion significantly decreased when the function of CFTR was attenuated by an inhibitor; somewhat interestingly, this occurred regardless of whether patients were diagnosed only with pancreatitis, or additionally (one patient only) with mild CF. Without ductal cells, or with a non‐porous membrane between compartments, this attenuation effect was not observed. The OoC thus revealed the direct functional coupling of ductal cells and hormone‐secreting cells, and might pave the way for understanding heterogeneity in pancreatic disorders. Pointing the way toward more comprehensive diabetes models, Bender et al. co‐cultured intact human islets (isolated post‐mortem) with primary EC progenitors and lung fibroblasts in a fibrin hydrogel [251]. Over 5–7 days, a vascular network self‐organized in the hydrogel, including adjacent to (but not penetrating) the islets, facilitating perfusion. The islets preserved their native cytoarchitecture, with glucose responsiveness and insulin secretion dynamics comparable to in vivo (though confounded by well‐known islet heterogeneity). Crucially, when perfusing alloreactive T cells—derived and induced from major histocompatibility complex‐*mis*matched blood—the researchers demonstrated islet invasion and cell damage, akin to the autoimmune dysfunction in type 1 diabetes.

The potential of even more advanced OoC architectures for pancreatic modeling has been demonstrated with a number of cancer models, where key roles of vascular perfusion and fibroblast‐dependent tissue stiffness were also revealed [252, 253]. Together with the recent patient‐derived pancreas models, these findings support the value of dynamic in vitro systems that recapitulate physiological interactions. Yet, the integration of iPSC‐derived pancreatic cell types with relevant cellular interaction partners in OoC remains limited, and establishing such systems could open new avenues for modeling pancreatic disorders.

### 3.8 Urinary Tract

The urinary system is responsible for fluid and electrolyte homeostasis and filtration within the body, involving complex reciprocal actions of the kidneys, where waste is filtered out of the blood and forms urine, and the downstream bladder, where urine is stored [254]. We discuss both organs and the related ducts in separate subsections.

#### 3.8.1 Kidney

The kidney is composed of functional units called nephrons. Each nephron consists of two parts: the proximal renal corpuscle for waste filtration and the renal tubule for further processing and transport [255]. The function of the renal corpuscle relies on fenestrated glomerular ECs, the glomerular basement membrane, and epithelial podocytes [256]. The podocytes provide structural support, regulate EC growth and function, and form interdigitated structures with their foot projections known as slit diaphragms [257]. These are primary contributors to the corpuscular filtration function, preventing macromolecules from passing through the barrier. The fenestrations between the glomerular EC are significantly larger, but the glycocalyx covering these cells ensures an active role of the ECs in the filtration process. The renal tubule is comparatively simple, consisting of a single‐layer epithelium. However, the tubule can be divided into at least 14 distinct sections, comprised of at least 16 distinct types of epithelium involved in different reabsorption and secretion processes [258]. Most prominently, the proximal tubule reabsorbs up to 60% of the substances filtered out by the glomerulus, from salts to glucose to amino acids [255]. Henle's loop and the distal tubule recover water, concentrating the urine, and exchange ions to maintain systemic osmotic balance [259].

Primary kidney cells have good availability, including, for example, transplantation discard materials [260]. More prominently, as many renal diseases involve the filtration barrier being compromised, an increased amount of primary glomerular ECs and podocytes end up in the urine of patients suffering from them, allowing for entirely non‐invasive isolation [261, 262]. Still, protocols for deriving nephron progenitor cells from iPSCs and differentiating them into renal iPSC‐epithelium and iPSC‐podocytes have also been developed [263]. These iPSC‐derived renal cells could be valuable to study rare genetic kidney diseases such as Alport syndrome, Dent's disease, Bartter syndrome, etc., as well as an equally large number of familial/congenital kidney disorders with unknown genetic causes [263, 264].

In 2017, Musah et al. published the first microfluidic model of the renal corpuscle in a vertical/membrane OoC [265]. iPSC‐derived intermediate mesoderm cells were cultured on one side of the membrane, the differentiation into mature iPSC‐podocytes being completed in situ. Primary human glomerulus ECs were seeded on the other side of the membrane. The researchers demonstrated that the combined application of cyclic strain—mimicking the blood flow‐related in vivo pulsations of the glomerulus [266]—and fluid perfusion improved podocyte differentiation. Specifically, they observed increased numbers of podocyte processes making contact with the endothelium, and increased podocyte secretion of collagen IV and VEGF‐A, an important factor in in vivo glomerulus development. Roye et al. built on these initial results with a fully isogenic iPSC‐derived model using commercial vertical/membrane chips [267]. The ECs used were, however, not specifically glomerular, and in neither study does the endothelium show the fenestrations that are present in vivo. Second, both studies use a thick, porous PDMS membrane to separate the two cell types, hindering the formation of slit diaphragms. Tabuchi et al. presented an effort that might remedy this shortcoming in the future using a lateral/hydrogel architecture [268]. Their design allowed for a compartmentalized culture of iPSC‐podocytes and a HUVEC endothelium, but they were not able to demonstrate podocyte foot process growth, potentially due to the thickness of the fibrin gel or lack of mechanical stretch included in this model. Petrosyan et al. moved toward patient‐specific modeling, relying instead on primary cells [260]. Using a commercial lateral/hydrogel system, they cultured glomerular ECs and podocytes directly on a collagen I gel. Healthy models were exposed to the serum of membranous nephropathy patients, resulting in podocyte and EC injury as well as compromised barrier function. The researchers also employed podocytes derived from a patient with Alport syndrome, a condition where a genetic mutation results in the production of a defective glomerular basement membrane. This resulted in a reduced ability of the barrier to retain albumin, a clinical marker of kidney damage. The ECs used in this model were not isogenic, being isolated from kidneys post‐mortem.

So far, distal tubule sections have been less popular subjects of modeling, with OoCs relying almost exclusively on non‐human cells or cell lines [269]. Bernardi et al. only recently published a preliminary report on the first such OoC using human primary cells [270]. Using a commercial lateral/hydrogel chip, dissociated primary organoids (differentiated toward distal tubule) were cultured against a collagen ECM gel. Barrier function and maturation markers were improved compared to static cultures, and active sodium transport was demonstrated. Proximal tubule OoCs are more common, including Gijzen et al.'s immunocompetent model in a customized version of that same lateral/hydrogel chip (Figure 3e) [119]. Separated by a collagen I matrix, they seeded the two respective microfluidic channels with a proximal tubule epithelial cell line and with a co‐culture of HUVECs and primary monocytes. Renal inflammation was induced with complement‐activated serum, leading to epithelial changes, cytokine release, and donor‐dependent monocyte migration, which could be mitigated by preclinical immune‐modulatory compounds. Toward iPSC‐based modeling, Banan Sadeghian et al. mixed the aforementioned immortalized cells with proximal tubule cells isolated from iPSC‐derived kidney organoids, co‐cultured with HUVECs on the opposing sides of a vertical/membrane OoC [271]. This triple co‐culture formed a more functional barrier than any dual co‐culture, showing improved filtration, reabsorption, and stable P‐glycoprotein transporter activity. The same group subsequently focused on only iPSC organoid‐derived proximal tubule cells in the same OoC [272], paralleling earlier work by Aceves et al. in a dual‐channel needle‐templated gelatin–fibrinogen hydrogel [273]. Both groups were able to show improved solute carrier protein polarization compared to immortalized cells in the respective same OoCs. They further were able to demonstrate more physiologically relevant nephrotoxicity response, prophylaxis, and transport using a selection of known toxic chemicals, inhibitors, and drugs.

There have been efforts to combine glomerulus models and proximal tubule models in order to realize a full nephron‐OoC. In 2021, Zhang and Mahler published such a combined model by non‐linear interconnection of two OoCs using tubing [274]. Their glomerulus OoC hosts HUVECs and immortalized human podocytes on a nanoporous membrane, under somewhat unique and not entirely physiological perpendicular perfusion. This OoC is connected in‐between the vascular‐side outflow and urinary‐side inflow of a vertical/membrane OoC, custom‐made in plastic and hosting both HUVECs and a proximal tubule cell line. The researchers demonstrated improved retention of albumin in the tri‐culture model, compared to a similar system with only proximal tubule cells. Qu et al. pursued integration of both barriers on a single OoC, but relying fully on animal cells [275]. Their more physio‐mimetic design consisted of a vascular layer and a renal layer, each split into two chambers with a membrane vertically separating relevant ECs and epithelium: the first chamber glomerular, the second chamber tubular. They were able to demonstrate and study the nephrotoxic response to two chemotherapy drugs. As an orthogonal approach relying on biological rather than engineered complexity, Kroll et al. developed a perfusable kidney‐OoC [276]. They combined hESC‐derived kidney organoids and a HUVEC‐endothelialized macrovessel within a gelatin–fibrin ECM, allowing organoid‐endogenous endothelial cells to establish a functional barrier and form lumen‐on‐lumen anastomoses supporting perfusion. Their model more comprehensively mimics native kidney microenvironments and holds promise for drug screening and modeling vascular damage in kidney disease.

iPSCs are increasingly being used in renal disease modeling outside of microfluidic contexts. For example, Mae et al. developed a functional model of autosomal dominant polycystic kidney disease, a monogenic disorder where renal cysts develop predominantly on the collecting duct, by producing collecting duct organoids from CRISPR‐edited iPSCs with the mutation causing the disorder [277]. Tanigawa et al. used patient‐derived iPSCs for an organoid model of congenital nephrotic disease, and observed the impaired formation of slit diaphragms in organoids formed with these cells [278]. Translating such capabilities into microfluidic platforms with demonstrable advantages in recapitulating physiological human kidney function represents a promising avenue for future pathophysiological research.

#### 3.8.2 Bladder and Associated Ducts

After production in the nephrons, urine flows from the collecting ducts through the ureters to the bladder and continues on through the urethra. These parts of the urinary tract are largely characterized by a lining of transitional epithelium or urothelium, supported by connective tissue and SMCs [279]. The urothelium is formed by three cell types: basal cells with urothelial progenitor function, located at the luminal basement membrane; intermediate cells with a high proliferative capacity, typically forming multiple layers and regenerating injured urothelium; and fully differentiated superficial umbrella cells, restricting diffusion of water and solutes at the apical surface [254]. Several biomechanical forces are crucial for the whole urinary system. Whereas rapid blood flow and hydrostatic pressure achieve an efficient filtration at the glomeruli as described above, peristaltic contraction of the ureters ensures unidirectional flow into the bladder. Accumulating urine distends the bladder, expanding the tight junctions of umbrella cells, activating stretch receptors, and ultimately triggering detrusor muscle contraction to void urine through the urethra, with tight junctions rapidly returning to their sealed configuration [254, 279].

Available primary tissue is somewhat commonly obtained from procedures such as tumor resection or bladder removal performed in the context of cancer or bladder disease [280]. Highlighting the hereditary component of urothelial cancer, biopsy‐derived spheroids have demonstrated genetic and histopathological features of the original tumor; in a bladder‐shaped microfluidic device, responses of these spheroids to chemotherapeutic agents correlated with clinical outcomes in patients [280]. Lifetime risk is also heritable for urinary tract infection [281], and epigenetic alterations in urothelial cells induced by *E. coli* can enhance susceptibility to recurrent infections [282]. Despite the genetic relevance, iPSC‐derived urothelial cells from patients have, to our knowledge, not yet been used to investigate the disease background. Still, directed iPSC differentiation gave rise to urothelium with characteristic stratification that further matured in a Transwell system where it exhibited viable barrier function [283].

Uropathogenic *E. coli* bacteria, the most frequent cause of urinary tract infections [254], can proliferate as intracellular bacterial colonies in the superficial umbrella cells [284]. Intracellular bacteria may be better protected from immune surveillance and antibiotic treatment, but analyses of dynamic reactions of these bacterial communities and the host cells to antibiotics are fraught with complications. Sharma et al. aimed to investigate the dynamic bacterial persistence in a bladder‐OoC [284]. Primary human bladder microvascular ECs and epithelial cells were cultured in the two chambers of a commercial vertical/membrane chip, respectively, perfused with media and dilute urine. Application and release of linear strain were designed to mechanically resemble the filling and voiding of the bladder, which interestingly enhanced the bacterial burden observed after infection with *E. coli*. The rapid recruitment of primary neutrophils from the vascular bottom channel to the infected epithelial cells did not avert the formation of intracellular bacterial communities. These were subsequently able to reinfect the tissue in‐between intermittent antibiotic treatment, the OoC thus providing valuable insights into the dynamic nature of bacterial resistance and persistence in the face of both immune responses and antibiotic treatment.

Bioprinted bladder cancer‐OoCs featuring layered structures of cancerous bladder epithelial cells, fibroblasts, and ECs (all cell lines) illustrate one direction for further development [285, 286]. These models were tested as tools for predicting immunotherapeutic outcomes, with the addition of monocytic cells and T cells expected to further enhance readouts [287]. Also leveraging biomaterials, Hou et al. applied adjusted matrix stiffness of a hydrogel to better match the mechanical properties of the human urinary tract in their bladder‐OoC [288]. After selecting a smooth surface morphology and a moderate matrix stiffness to promote cell adhesion, viability, and differentiation, implementing physiological pulsatile and periodic fluid shear stress further enhanced differentiation and maturation of their human urothelial cell line.

Advancements in bladder‐OoC technology, which incorporate suitable (patho)physiological mechanical forces, provide a strong model foundation. These systems have proven valuable for studying, for example, infectious diseases and tumor behavior. Future integration of patient‐derived primary or iPSC‐derived cells should further enhance physiological relevance and support personalized approaches to studying bladder diseases and evaluating therapeutic strategies.

### 3.9 Reproductive Systems

For both the male and female reproductive tracts, barriers separate the reproductive cells from the rest of the body. Additionally, the fetal‐maternal interface and the blood‐milk barrier will be discussed. Dysfunction of these highly specialized barriers can lead to infertility, pregnancy complications, and fetal/neonatal maldevelopment. Not all relevant cell types have iPSC derivation protocols available, and several pose unique challenges (e.g., germ cells, extraembryonic lineages, short lifetime). Beyond disease models, OoCs of the reproductive system could offer new insights into human fertilization, implantation, embryonic development, and maternal‐fetal and ‐neonatal exchange, which—due to the time‐constrained and often human‐specific biology alongside clear ethical limitations—are difficult to study in vivo.

#### 3.9.1 Testes

Within the testicles, spermatogenesis begins within structures called the seminiferous tubules, and continues in the epididymis, a coiled tubular structure where spermatozoa mature and are stored. The basement membrane of the seminiferous tubules hosts peritubular myoid cells (a type of SMC) on the outside, providing structural support and contractility [289]. The tubule lumen is host to Sertoli cells, which have a nurturing function for the germ cells maturing in direct contact with them. At the same time, Sertoli cell tight junctions regulate paracellular transport and act as an immunological barrier. This includes not only infection, but also, for example, preventing the development of anti‐sperm auto‐antibodies that can cause infertility in cases of disrupted barrier function (which may be congenital) [290, 291]. In the epididymis, spermatozoa gain the functions of swimming and fertilization [292]. Its tightly linked barrier epithelium mainly consists of principal cells, which excrete proteins and exosomes into the lumen. Narrow, clear, and apical cells are known to play a role in the acidification of the lumen, and clear cells also have a role in the absorption of material from the lumen. Surrounding peritubular SMCs are (partly) responsible for luminal fluid flow, which is likely to play a role both in epididymis and sperm maturation [293].

Primary Sertoli or epididymis cells have been used for in vitro models, but healthy donors are rare, and the yield from testicular biopsies is low [294, 295]. As a result, non‐human models have been popular in spite of biological limitations [294]. While Rodríguez Gutiérrez et al. were able to derive Sertoli‐like cells from iPSCs [296], to our knowledge no iPSC to epididymis differentiation protocols have been published. Progress on OoC remains equally limited; as with ovaries earlier, an initial attempt at microfluidic ex vivo culture of human testes fragments showed no significant difference compared to static culture [297] Yet both the Sertoli and the epididymis barriers could clearly benefit from the biomechanical control of OoCs in the study of male infertility. Moreover, the cause of roughly half of the cases of male infertility remains unclear, with genetic conditions thought to be likely [298]. Knockout models of various Sertoli barrier‐related genes have been found to lead to infertility in mice [299]. Personalized OoC of these barriers could thus be a valuable avenue for research.

#### 3.9.2 Accessory Male Sex Glands

Around the interface of the sperm duct and the urinary tract are located the seminal vesicles, prostate, and the bulbourethral (or Cowper's) glands. The seminal vesicles contribute a variety of components—notably fructose and prostaglandins—to the seminal plasma and are responsible for more than half of the ejaculate volume. They consist of convoluted, blind‐ending tubes of glandular epithelium that join the sperm duct. The bulbourethral glands secrete pre‐ejaculatory fluid that neutralizes the acidic environment of the urethra and provides lubrication. They are tubuloalveolar glands that exit into the urethra. Congenital defects—such as Cowper's syringocele or seminal vesicle hypoplasia—in these glands can lead to infertility or reduced fertility [300, 301, 302]. To our knowledge, no in vitro disease models have been published on the seminal vesicles or bulbourethral glands, nor any protocols for deriving relevant cell types from iPSCs.

Lastly, the prostate is an exocrine gland in the male reproductive system that secretes part of the seminal fluid. Prostate additions to the seminal fluid include proteins as well as significant levels of zinc and citric acid [303]. These are not strictly necessary for fertility, but do have a protective effect on the sperm cells [304]. The prostate epithelium consists of luminal cells, non‐secretory basal cells that act as stem cells for the luminal cells, and rare neuroendocrine cells [305]. At least one protocol of iPSC‐derived prostate tissues has been established [306]. The most common diseases of the prostate are benign prostatic hyperplasia and prostatic cancer [307], where models could be built using primary proliferative/cancer cells, harvestable through invasive biopsy [308, 309]. In one of the few OoCs in this realm, employing a vertical/membrane approach, Jiang et al. cultured immortalized primary basal prostatic epithelium and benign human prostate stromal cells in two respective compartments [310]. The model showed how androgen stimulation of the stroma could induce differentiation of the basal cells toward luminal cells. Taken together, it is clear that only limited effort has gone into the development of OoCs of the various glands of the male reproductive tract.

#### 3.9.3 Ovaries and Fallopian Tubes

Within the ovaries, each ovum develops inside a structure known as an ovarian follicle. The follicular barrier consists of a layer of epithelial‐like granulosa cells, separated from the external network of vascular capillaries by two distinct types of basement membrane [311]. The barrier cells ensure that the follicular fluid retains, for example, high levels of anti‐oxidants, and further supports ovum development when triggered by the relevant hormone, lutropin. The fallopian tubes or oviducts later provide a suitable microenvironment for fertilization and transport the developing embryo to the uterus [312]. The inner lining of the oviduct consists of both secretory and ciliated epithelial cells. The ciliary beating picks up the oocyte and transports it toward the uterus; it also causes flow in tubal fluid. Reduced ciliary activity can lead to an ectopic pregnancy, where the fertilized egg implants outside the uterus. The composition of the tubal fluid is regulated by the secretory cells, which secrete nutrients, growth factors, and antimicrobial agents. The fluid facilitates sperm transport, provides a stable environment for fertilization, and removes debris and non‐motile sperm [313]. Protocols for fallopian iPSC‐epithelium have been developed, and are of interest for studying high‐grade serous ovarian carcinoma, a cancer thought to originate from these cells [314, 315]. Fallopian iPSC‐epithelium could also be a useful cell source for modeling the effects of CF or primary ciliary dyskinesia on fertility [316]. Protocols for deriving oocytes and granulosa cells from iPSCs have been published as well [317, 318]. iPSCs from patients with familial forms of ovarian insufficiency and polycystic ovary syndrome have already shown promise in plate‐based in vitro research [319, 320]. Yet corresponding OoC models remain elusive. There have been some efforts on culturing and maturing ex vivo ovarian follicles [321], with one human‐derived attempt showing no difference between microfluidic or static culture [322]. Existing microfluidic models of the oviduct, meanwhile, were designed to study interaction of the oviduct with sperm and fertilization, and rely at best on animal cells [323, 324]. The compartmentalization and perfusion capabilities of OoCs thus hold untapped potential to shed further light on associated pathophysiological processes when combined with corresponding functional cells.

#### 3.9.4 Uterus

The uterine cavity is lined by the endometrium. During reproductive life, the surface layer of the endometrium is in a cyclic process of shedding (menstruation), regenerative proliferation, followed (after ovulation) by differentiation and secretion [325]. This cycle is regulated by steroid hormones, predominantly estradiol and progesterone [326]. The tight endometrial barrier comprises luminal epithelial cells; these further give rise to glandular epithelial cells forming secretory crypts that produce mucins to support uterine homeostasis and implantation receptivity. Underlying stromal cells contribute to endometrial remodeling, menstrual cascade propagation, and tissue breakdown, with a sub‐population of progenitor stem cells instrumental to the regeneration phase. Cell–cell communication between the epithelial and stromal cells of the endometrium is thus crucial for normal function [327]. On the outside of the endometrium is the myometrium, a layer of smooth muscle responsible for uterine peristalsis, which can in turn impact the endometrium. Uniaxial, cyclic tensile stretch has been found to increase the acquired contractility of endometrial stromal cells [328] and peristalsis‐like fluid shear was found to increase F‐actin filament production in endometrial epithelium [329].

Primary endometrium can be obtained from biopsies or curettage. An alternative, less invasive source of primary endometrium is menstrual blood, though this comes with the caveat that it mostly contains cells from the surface layer, not the basal layer, with derived cells exhibiting somewhat distinct behavior in vitro [330]. Protocols have recently been developed to derive endometrial cells from iPSCs, but they have not been applied to OoCs as of yet [331, 332]. We note that animal models are particularly limiting for studying the human endometrium, as the menstrual cycle is rare, occurring only among primates, a few species of bats, the elephant shrew, and the spiny mouse. Significant inter‐species differences in cycle regulation and endometrial physiology further restrict the applicability of these models to humans [333].

Endometrium‐OoCs are of special interest in the study of endometriosis, a debilitating condition where endometrial tissue grows outside of the uterus [312]. The underlying pathology of endometriosis remains unclear, with genetic predisposition, immune response, and environmental factors all playing a role [327]. Chen et al. developed an innovative (electrospray‐based) approach to encapsulate primary human endometrial stromal cells within the cellulose core of microparticles with an alginate shell [334]. These cell‐laden microcapsules were cultured in an array of chambers on a microfluidic chip, enabling uniform endometrial spheroid formation under perfusion. An integrated concentration gradient generator facilitated drug screening, with the researchers able to demonstrate patient‐specific drug sensitivities. Correlations with 12‐month clinical patient outcomes highlight the potential to guide personalized therapeutic interventions for endometriosis. Kapur et al. instead utilized a more typical—and more barrier‐focused—OoC approach based on needle‐templated collagen [335]. Lining it with (immortalized) endometriosis‐derived epithelial cells, they investigated the therapeutic benefits of oxidative stress‐inducing agents.

Some endometrium‐OoCs with greater biological or engineered complexity have also been developed without targeting specific disorders, but can serve to illustrate clear OoC advantages. Focusing on hormonal menstrual regulation, Gnecco et al. cultured primary uterine microvascular ECs and endometrial stromal cells on opposing sides of a vertical/membrane OoC, showing that vascular perfusion enhanced decidualization via induction of prostaglandin expression in the ECs [336, 337]. Ahn et al., conversely, pursued a five‐chamber lateral/hydrogel OoC [338]. Incorporating a self‐organized HUVEC vascular network along with cell line‐based endometrial stromal fibroblasts (in fibrin gel stromata) and epithelial cells, they were able to recapitulate hormone‐driven epithelial thickening, vascular remodeling, and increased permeability under contraceptive exposure. In addition, they demonstrated attachment of microbeads intended to represent (and of similar size to) embryos. These examples demonstrate the potential of OoCs for dynamic endometrium modeling and related research into, for example, endometriosis or complications in zygote implantation or pregnancy.

#### 3.9.5 Fetal–Maternal Interface

During pregnancy, the developing embryo is encapsulated in a fluid‐filled sac. The innermost part of the surrounding fetal membrane, the amnion, consists of a single layer cuboid epithelium and arises from the mesoderm concurrent with blastocyst implantation. The amnion is surrounded by a multilayered ECM providing structural support and containing fibroblasts and amniotic mesenchymal cells. The latter mediate the constant repair necessitated by damage incurred through shear stress, fetal movement, or cell senescence. Outside is the chorion, which becomes highly vascularized and, in close contact with the maternal decidua during pregnancy, develops long and complex villi in order to form the placenta [339]. The placenta is a transient organ that forms the barrier between the maternal and fetal blood circulations. Through this barrier, nutrients and waste products are exchanged. Maternal blood pools in the placental lobes, and compounds then move through three layers that make up the human placental barrier [340]: the syncytiotrophoblast cell layer, a thin layer of connective tissue, and the fetal vascular endothelium. Syncytiotrophoblasts are multi‐nucleated cells that arise from the terminal differentiation and fusion of chorionic cytotrophoblasts. The syncytiotrophoblast layer is the primary regulator of cross‐barrier transport in the placenta.

Understanding these transport mechanisms is important to predict how exposure to certain external factors, including both drugs and disease, may affect the fetus. Yet the fetal–maternal interface presents unique challenges in obtaining relevant cells. For some time, models of the placental barrier have been developed using choriocarcinoma‐derived cell lines and isolated primary placental cells to represent the syncytiotrophoblast layer [341, 342, 343]. Relevant biopsies may be performed for prenatal diagnostics, but do carry a risk of miscarriage (post‐natal availability, by comparison, is high). iPSC reprogramming for personalized prenatal modeling would have to rely on similarly obtained fetal cells, though isolation of rare fetal blood cells from maternal blood could provide an alternative [344]. Additionally, it is widely recognized that PSCs do not differentiate toward extraembryonic lineages in vivo; however, it has been found that human iPSCs can be directed toward early trophoblast lineages in vitro [345, 346]. This transdifferentiation can be enhanced by employing so‐called naïve iPSCs, which can either be derived directly during reprogramming, or converted from “standard” primed iPSCs through defined culture conditions [347, 348, 349]. Still, the timeline of reprogramming and differentiation is not favorable for truly individualized models of the fetal–maternal barrier, given its transient nature. iPSC models can still be a valuable tool, alongside primary cells, to more generally model barrier dysfunction and study genetic predispositions as well as cell–cell signaling defects in disorders such as preeclampsia, which may arise from malfunction or malformation of placental vasculature [350, 351, 352].

Li et al. developed a notable placental barrier model from iPSC‐trophoblast progenitors by seeding them directly on top of HUVECs and fibroblasts (from a lung cell line) in a static Transwell‐like system [353]. They found that drug transport in their system more closely resembled that of an earlier perfused ex vivo placenta model [354] than a cancer cell line monoculture. Although “microvilli‐like features” were observed, previous research has shown that shear stress improves microvilli formation (at least for that same cancer cell line) [355], suggesting that mechanical stimulation could further enhance iPSC‐based models. More recently, by similarly employing a vertical/membrane OoC, Delon et al. further emphasized this in a co‐culture of HUVECs with the trophoblast cell line [356]. Under pumpless recirculating flow, they found not only microvilli formation but also enhanced syncytialization markers, barrier integrity, and nutrient transporter expression. By contrast, Deng et al. cultured iPSC‐trophoblast progenitors in an OoC, with the cells embedded in matrigel and perfused on top [357]. They demonstrated improved maturation and differentiation of their organoid‐like constructs, morphologically more similar to primary tissue than static cultures. Wang et al. subsequently demonstrated how such organoids (here from primary trophoblast progenitors) could be further enhanced and utilized by co‐culture with HUVECs, placed on opposing sides of a vertical/membrane OoC [358]. They were able to demonstrate, for instance, microvilli formation on the organoids, something that was lacking in organoids alone both here and in Deng et al.'s study. The lack of accessible trophoblast formation, however, limits both systems' applications. This was addressed by Cao et al., employing the same OoC as Wang et al. [359]. Instead of organoids, they directly cultured primary trophoblast progenitors on the membrane, with HUVECs and media perfusion underneath. They observed differentiation into a bi‐layer of cytotrophoblasts and syncytiotrophoblast, creating a functional placental barrier OoC. With the further addition of a monocyte cell line, they proceeded to study inflammatory response and fetal coupling upon maternal nanoparticle exposure. Finally, the most biologically comprehensive, albeit static, attempt at a fetal–maternal OoC was undertaken by Vidal et al. [360]. Incorporating seven relevant cell types across as many collagen I or IV‐coated compartments, interconnected by narrow microchannels, it sought to approximate second‐trimester placenta with a combination of primary and immortalized cells. With this platform, they were able to study cell type‐specific and organ‐holistic endocrine disruption and (anti‐)inflammatory response in response to relevant chemical exposure.

Modeling the fetal membranes is especially of interest for the study of preterm prelabor rupture of the fetal membranes. This is syndromic in nature, with inflammatory cues (e.g., from smoking) and genetic predispositions in both mother and fetus playing a role [361]. Based on postnatal primary cells, Richardson et al. published an amnion OoC, consisting of a central mesenchymal chamber, connected to a surrounding epithelial chamber by collagen IV‐filled microchannels [362]. They proceeded to expose monocultures or cocultures to cigarette smoke extract. The researchers observed complex differential effects on migration and transition (epithelial–mesenchymal and vice versa), crucial processes in amnion maintenance. The same group also studied the effects of the same intervention on the maternal–fetal interface by combining primary amniotic epithelial cells instead with decidual cells [363]. Cultured in the two respective chambers of a vertical/membrane OoC, they found increased inflammatory response compared to a Transwell system. These two OoCs, however, lacked dynamic perfusion. In vivo, the amniotic fluid subtly rocks back and forth and the amniotic epithelium is exposed to low levels of undulating shear stress. Kim et al. sought to investigate this using the aforementioned amnion‐OoC, with the outer epithelial compartment connected to a syringe pump (Figure 3f) [120]. Perfusion did lead to microvilli formation, which is observed on in vivo amniotic epithelium. The researchers somewhat surprisingly conclude that (given some other readouts being unaffected) that flow is not required in amniotic models; lacking in vivo data, it also remains unclear whether the applied (blood vessel‐like) shear rate was physiologically relevant. The complete fetal‐maternal interface (i.e., placenta and fetal membranes) was recently combined into a single OoC model by Safarzadeh et al. [364]. Closely approximating the design employed by Vidal et al., their laterally interconnected chambers hosted seven distinct cell types (primary and cell line) to recapitulate both the placental interface and amniochorionic interface in the same device side to side. They demonstrated the utility of the system for modeling drug transportation and reaction to inflammatory factors, but acknowledge shortcomings in cell source and lack of active perfusion.

Due to its importance in fetal–maternal exchange, most fetal–maternal interface modeling efforts have focused on the placenta, with fetal membrane models being a more recent development. Research into both tissues is complicated by the partially fetal origin of relevant cell types, entailing invasive and risky harvesting. Still, there is unrealized promise in studying relevant disorders post‐natally by means of iPSC‐derived OoC.

#### 3.9.6 Vagina and Cervix

The cervix and vagina form the birth canal, separating the uterine cavity from the exterior. The cervical canal is lined by the endocervix, a glandular, columnar epithelium [365]. At the vaginal end, this transitions into the ectocervical stratified squamous epithelium. The intermediate “transformation layer” is home to reserve cells, bi‐potent cells that can differentiate to both columnar and squamous epithelia [366]. The vaginal wall is similar to the ectocervix, consisting of a mitotically active basal layer, a suprabasal layer of cells that undergo terminal differentiation to flattened, cornified cells (not unlike the epidermis), which form the outermost layer, the superficial stratum corneum [367]. The cells of the stratum corneum are loosely connected, devoid of nuclei and organelles, and contain glycogen deposits. Together with the cervicovaginal mucus—produced predominantly in the endocervix, from where it migrates along the cervical canal to the vaginal cavity, serving also a clearance function—this provides a suitable environment for the vaginal microflora. This microflora consists predominantly of *Lactobacillus* species, their lactic acid production lowers the cervicovaginal pH level, in turn protecting against other microbial pathogens [367, 368]. During pregnancy, a cervical mucus plug protects the uterus from vaginal pathogens. Patient‐specific in vitro models of the cervicovaginal tract are of interest for the study of genetic predisposition to cervical cancer or vaginosis, but also with regard to, for example, CF [369, 370, 371]. Primary cells of the cervicovaginal tract are readily available, though with varying levels of invasiveness. To our knowledge, no protocols for deriving vaginal epithelium from iPSCs have been published. Sato et al. did publish a protocol for deriving cervical reserve‐like cells from iPSCs, which could be used to provide both the columnar and squamous cervical epithelium [372]. Such cells have, as of yet, not been applied to OoC models.

OoC models of the cervicovaginal tract are a fairly recent development. Mahajan et al. employed a commercial vertical/membrane chip, seeding primary human vaginal epithelial cells and uterine fibroblasts on the opposing membrane sides [373]. They observed spontaneous epithelial differentiation into a multi‐layered, stratified structure reminiscent of vaginal walls. *Lactobacilli* were able to colonize the epithelium, showing physiologically relevant levels of lactic acid production and downregulation of inflammatory cytokines. The same group later proceeded to model cervix, employing instead primary cervical epithelium (mixed endo‐ and ectocervix) and cervical fibroblasts, respectively [365]. Focusing on the specific advantages provided by control over perfusion, they found improved barrier formation compared to static Transwells, as well as more physiologically relevant mucus composition. Mucus production was moreover responsive to menstrual cycle‐mimicking hormones. They moreover observed that continuous flow (in both channels) upregulated genes associated with an ectocervical phenotype, whereas changing to intermittent flow in the cervical compartment upregulated endocervix‐associated ones (a result that bears translation back into their vaginal model, where they had employed the latter perfusion approach). Both vaginal and cervical OoCs showed inflammatory responses to unfavorable bacteria (though co‐culture with *Lactobacilli* was not investigated). Interestingly, Gutzeit et al. most recently demonstrated the protective role of cervical mucus in vaginosis by collecting mucus produced in the cervix‐OoC and introducing it in the vagina‐OoC [374].

The opportunities offered by cross‐reproductive tract coupling were further highlighted by Tantengco et al., albeit with immortalized cells only [375] The researchers developed a cervicovaginal OoC featuring six distinct interconnected culture chambers and cell types, mirroring the strategy they would later employ for fetal‐maternal‐OoCs (cf. Safarzadeh et al. and Vidal et al. [360, 364]). In conjunction with a somewhat simpler fetal–maternal‐OoC loosely based on their earlier amnion‐OoC (cf. Richardson et al. [362]; expanded to chorion and decidua), they showed that *U. parvum*‐infected ectocervix can spread exosomes along the tract and cause inflammation in the decidua, but not the fetal membrane. This, along concurrent murine validation, suggested that *U. parvum* alone is not sufficient to cause pre‐term birth (with which it had previously been clinically correlated). These efforts toward vaginocervical OoCs successfully incorporate the distinctive epithelial structure as well as its microbial interactions. Such models could offer valuable insights in, for example, pre‐term birth causing disorders or the effects of CF on female reproductive tract.

#### 3.9.7 Mammary Glands

The alveoli and adjoining lactiferous ducts consist of two cell layers: an inner layer of luminal epithelium and an outer layer of myoepithelium in contact with a basement membrane. The alveolar luminal cells are responsible for milk production. The myoepithelium squeezes the produced milk into the ducts [376, 377]. The alveolar luminal epithelial cells express many basolateral transporters and channels in order to absorb the many nutrients necessary to produce milk from adjoining vasculature, including glucose, fatty acids, and amino acids. This epithelium forms tight‐junctions to prevent the paracellular leakage of blood and milk. The permeability of this blood–milk barrier is variable, becoming less permeable during lactation. Breast cancers, for which tumors can be excised from patients, are currently the most modeled disease in this context and also hold the most prominent example of a genetic cancer risk factor, that is, breast cancer genes 1 and 2. Furthermore, several genetic variations are known to cause changes in milk composition, which can cause nutritional deficiencies or impaired gut microbiota formation in infants [378].

In these cases, iPSC‐derived cells can be valuable for disease modeling, and protocols for deriving mammary tissue from iPSCs have been published [379, 380]. Primary cells also remain an option here, as organoids suitable for study can be obtained from normal breast tissue [381, 382]. Neither have been translated into OoC models, in spite of the presumed importance of biomechanics. Cho et al., for instance, studied a mammary epithelium cell line cultured inside a (static) lateral/hydrogel OoC [383]. They showed that the stiffness, density, and surface protein makeup of the collagen I matrix had substantial impact on epithelial ECM secretion, hydrogel invasion, and barrier leakage. More recently, Buchholz et al. introduced a 3D mammary duct OoC based on rapid volumetric printing of arbitrary channels inside gelatin methacryloyl [384]. Seeding a mammary epithelial cell line, they demonstrated improved organization, polarization, and barrier function under pulsatile flow (and associated stretch) compared to 2D or static 3D cultures; they further demonstrated a proof‐of‐concept co‐culture with HUVECs in an adjacent channel, and MSCs mixed into the hydrogel. In the future, such integrated models, particularly based on iPSCs, could enable studying human milk composition and the variability therein, and the effects this has on infants, in more physiologically relevant settings.

### 3.10 Thyroid

The human endocrine system consists of the thyroid, parathyroid, pituitary, pineal, and adrenal glands, in addition to endocrine functions of the testes, ovaries, thymus and pancreas. Most of the endocrine glands consist of secretory cells in a vascularized stroma, with the relevant endothelial barrier not particularly specialized. For the purposes of this review, only the thyroid is thus of further interest.

The thyroid is responsible for the production of the critical metabolic hormones thyroxine and triiodothyronine. Their production occurs in epithelial thyrocytes that are organized in a single layer around a fluid‐filled lumen [385]. These thyroid follicles are in turn embedded in a highly vascularized interstitium. The polarized thyrocytes are specialized in uptake of the rare but vital iodide, and transport it to the follicular lumen. There it is incorporated in thyroglobulin as a hormone‐precursor and subsequently transported and processed back toward the bloodstream [386]. Disorders of the thyroid manifest in either hypothyroidism (partial or complete loss of thyroid hormone production) or hyperthyroidism (excessive thyroid hormone production). Both conditions are a part of pathologies with genetic risk factors, such as Hashimoto's thyroiditis, congenital hypothyroidism, or Graves' Disease (hyperthyroidism).

Primary cells are readily available from cancer biopsies and may have utility as screening tools for personalized treatment [387]. Healthy thyrocytes, however, have a very slow turnover: they are estimated to only divide once every 10 years [388]. Thus, efficient protocols to derive thyrocyte‐like cells from iPSCs have been sought [389]. Progress has recently been made with gel‐embedded 3D cultures that self‐organize to form follicles and are capable of producing thyroxine [390, 391].

OoC models targeting the specialized thyroid barrier function remain rare, with most published works instead “simply” incorporating thyroid follicles in multi‐OoC systems [392, 393]. Carvalho et al. recently attempted to better recapitulate the thyroid hemodynamic microenvironment in a custom polycarbonate OoC [394]. They cultured *murine* stem cell‐derived thyroid follicles in matrigel, placed at the bottom of a microfluidic chamber (employing a removable carrier and PDMS gaskets, allowing for facile assembly and endpoint analysis). Perfusion over the top of the gel (at relatively low rates) allowed them to recapitulate blood‐like transport without exposing follicles to non‐physiological shear in the absence of an endothelial barrier. The authors were able to demonstrate significantly increased thyroxine accumulation in the lumen compared to static conditions, though they neglected to quantify secretion.

Due to the profound effects of thyroid hormones on virtually all tissues in the human body, a personalized human version of such a combined organoid‐OoC approach could be valuable for systemic disease models.

### 3.11 Multi‐Organ‐Chips

The evident importance of physiological links not just within but also between different barriers and organs necessitates reconstructing these connections as well. One approach to multi‐OoC coupling is to aspirate, transfer, and mix effluents between individual OoCs in an in vivo–like sequence. An early implementation of this, with media actually shipped between different research labs, connected OoCs of jejunum, liver, kidney, and BBB [395]. With models spanning the full range of cellular origins (lines, primary, iPSC‐derived) and architectures (Transwell, vertical/membrane, tubular hydrogel), they succeeded in recapitulating clinical absorption, distribution, metabolism, and excretion (ADME) for three reference compounds. Novak et al. improved upon this type of approach by using a pipetting robot to fluidically couple up to eight different OoCs [396]. Although highly modular, scalable, and adaptable, a tighter and more controlled coupling can be achieved by employing microfluidic interconnects (discussed further also in Section 3.12), or by fully integrated multi‐OoCs, which have been preferred in the more specific disorder models reviewed below.

**FIGURE 4:**
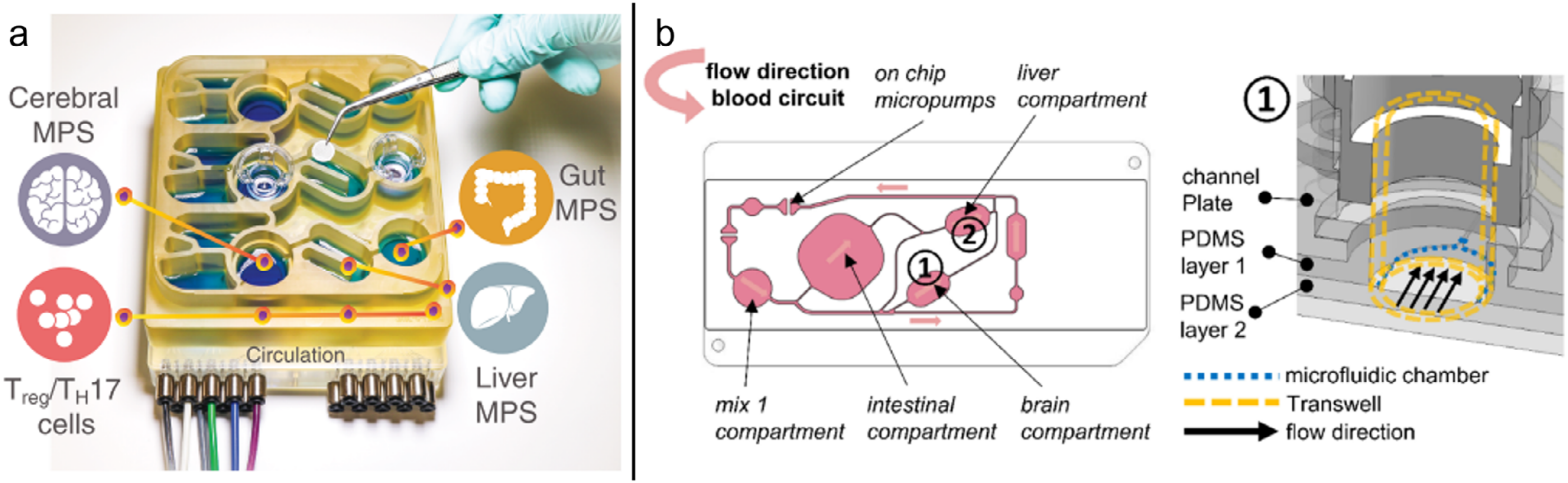
Multi‐Barrier OoCs. (a) Gut–Liver–Brain. A central liver compartment hosts hepatocytes and Kupffer cells on a lobule‐mimicking disc (see tweezers) and can be connected to two other Transwell‐based barriers/organs. The common circulation is enabled by plate‐integrated fluidics and pneumatics based on polyurethane membranes. Adapted under the terms of the CC BY‐NC license [397]. Copyright 2021, Trapecar et al. (b) Gut–Liver–Brain. Here, fully isogenic cell populations are hosted in a commercial multi‐OoC, with a surrogate blood circuit (pink; based on PDMS membranes and channels) connecting Transwell‐style compartments hosting intestinal and liver epithelial barriers, as well as a brain compartment (zoomed view on right) with blood–brain barrier iPSC‐endothelium exposed to the flow and iPSC‐neurospheres on top. Adapted under the terms of the CC BY license [400]. Copyright 2022, Koenig et al.

For a physiomimetic model of early‐onset Parkinson disease, Trapecar et al. impressively connected gut, liver, and brain—each contained in Transwell inserts—via a shared millifluidic continuous‐circulation platform (Figure 4a) [397]. The gut consisted of dissociated colon organoid epithelium, with blood cell‐derived macrophages and dendritic cells on the reverse side; micro‐structured liver scaffolds were seeded with hepatocytes and Kupffer cells; the common circulating medium contained two populations of blood‐derived T cells. Whereas all the above were from various healthy donors, the cerebral Transwell hosted neurons, astrocytes, and microglia derived from a mutation‐carrying Parkinson's disease iPSC line (or gene‐corrected control). Enhanced maturity of neurons, astrocytes, and microglia influenced by gut and liver cells was displayed in this multi‐OoC. Conversely, exposing the gut to microbiome‐associated short‐chain fatty acids altered gene expression globally, and, in the cerebral Parkinson's model specifically, augmented pathology‐associated disease pathways. The displayed interactions of immune cells and fatty acids can serve as references for future studies where also appropriate EC barriers are included. In a prior study, the same group used a largely analogous system (excluding the brain compartment) to model ulcerative colitis using relevant colonic donor tissues or healthy controls [398]. Interestingly, exposure to fatty acids either alleviated or exacerbated disease severity depending on T cell activity. During T cell‐mediated responses, fatty acids exacerbated inflammation, leading to gut barrier disruption and liver injury. Yet the non‐autologous nature of these multi‐OoCs remains a limiting factor. This was most comprehensively addressed by Ramme et al., who combined Transwell‐style intestine, liver, kidney, and brain on a single, commercial multi‐OoC perfusion platform [399]. All cells were predifferentiated from the same healthy iPSC line. The resulting spheroids/organoids (with only the kidney cells dissociated prior to seeding) were maintained on the multi‐OoC in a common medium without tissue‐specific growth factors for over two weeks. Advanced maturation of intestinal and liver tissues was observed over time. However, gene expression patterns of the kidney cells did not match those of fetal or adult kidney, indicating limitations in cell type purity. Together with an intestine relying on intact organoids, and a lack of ECs, the multi‐OoC showed insufficient formation of functional biological barriers (adversely impacting also neuronal maturity). Koenig et al. later employed the same isogenic multi‐OoC approach, though restricted to liver and brain, with BBB iPSC‐ECs on the reverse side of the iPSC‐neurospheres (Figure 4b) [400]. While they unfortunately did not revisit the question of neuronal maturation, it allowed the researchers to measure BBB permeation and metabolite distribution. Shinohara et al. established co‐cultures of hepatocytes (from humanized chimeric mice) with iPSC‐intestinal epithelium in a different multi‐OoC platform, also built to accommodate Transwell‐style inserts [401]. The directional, pneumatically driven perfusion of the multi‐OoC increased liver functions such as the expression of liver‐specific genes, albumin production, and cytochrome activity compared to mono‐cultures.

Most pathological developments affect more than one tissue or organ through complex biochemical, immunological, and mechanical pathways, often critical for understanding disease progression and therapeutic effects. Multi‐OoCs are well‐positioned to recapitulate this in a controlled and physiologically‐relevant manner. Fully autologous primary and/or iPSC‐derived systems carry the promise of moving the field closer to personalized, patient‐specific disease modeling and drug testing. Continued integration of more functional vascular, lymphatic, and immune systems, as well as moving beyond Transwell‐style multi‐OoCs to improve biomechanic and biochemical microenvironments, will be essential for advancing the physiological relevance and translational impact of multi‐OoCs.

### 3.12 Technology

Effective OoCs require thoughtful integration of disparate technologies, such as microfabrication, material/surface engineering, as well as sensors and actuators. Whereas our other sections have largely focused on state‐of‐the‐art biological components and their application, in this section, we aim to highlight technological limitations and relevant developments that—if integrated—could open up further possibilities for insight and impact.

#### 3.12.1 Engineered Interfaces

**FIGURE 5:**
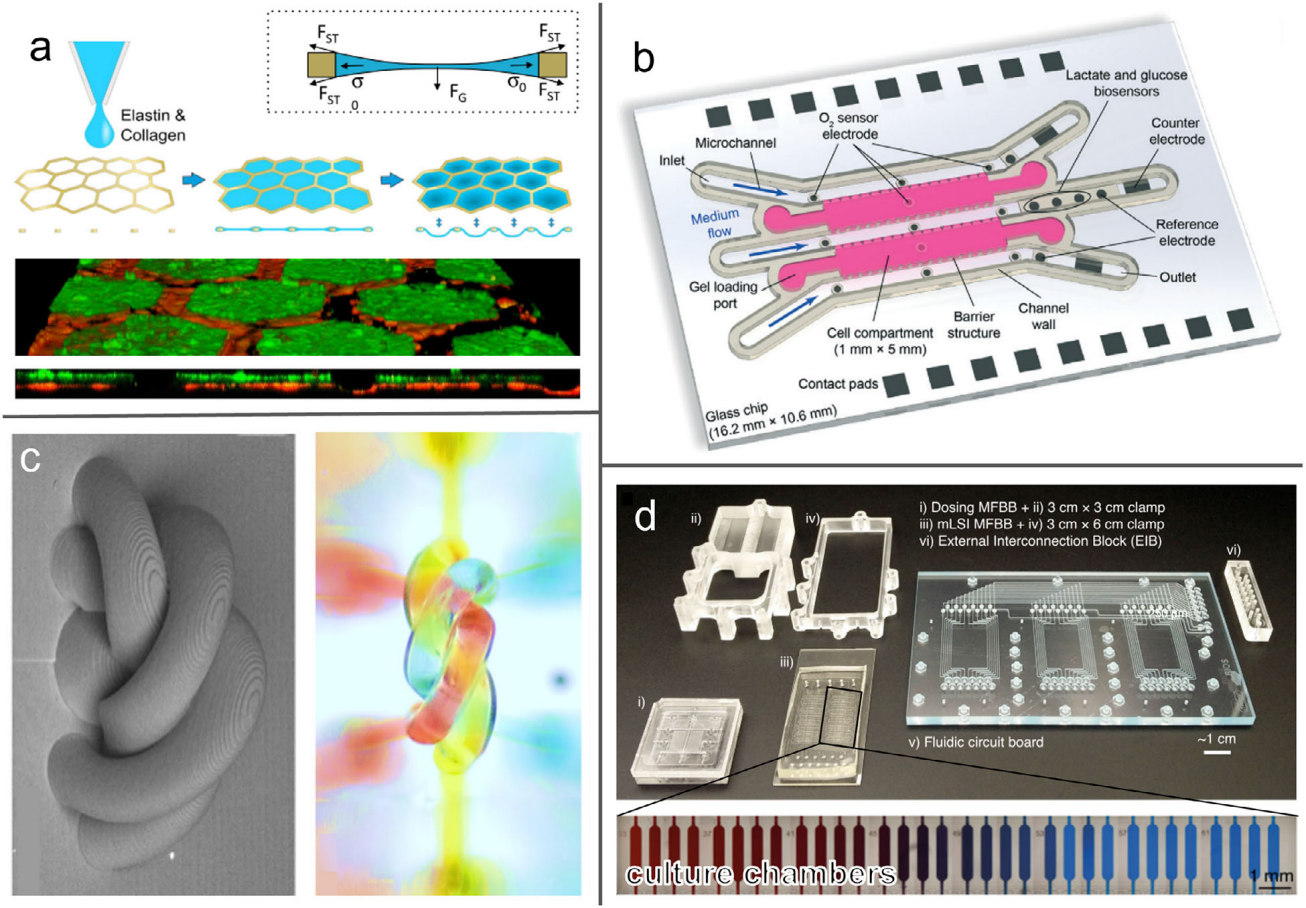
Examples of recent technological innovations. (a) Basement membrane‐like interfaces and radial stretch. Collagen–elastin solution self‐assembles into a membrane array inside a hexagonal gold mesh (260 μm pores), and can be hydraulic pressure‐actuated to apply breathing‐like homogenous out‐of‐plane stretch. Confocal reconstructions show the membrane populated with alveolar epithelium (green) and endothelium (red). Adapted under the terms of the CC BY license [407]. Copyright 2021, Zamprogno et al. (b) Biochemical monitoring. The sensor‐integrated chip comprises two cell compartments (pink), with electrochemical microsensors for oxygen, lactate, and glucose placed in the adjacent microchannels. Adapted under the terms of the CC BY license [410]. Copyright 2022, Dornhof et al. (c) 3D‐printed fluidics. Examples of 3D intertwined channels visualized by electron (left) or brightfield (right; with channels perfused by food colorants) microscopy. The monolithic process also allows for addition of micro‐porosity to the vessels (not shown). Adapted under the terms of the CC BY license [411]. Copyright 2025, Xu et al. (d) Standardization & Modularization. Range of ISO‐compliant chip components created as part of the Translational Organ‐chip Platform (TOP), including a main fluidic circuit board and connectors (v, vi) as well as modules for dosing (i) and highly parallel cell culture (iii and zoom‐in) that interface with it. Adapted under the terms of the CC BY license [412]. Copyright 2020, Vollertsen et al.

As discussed in the introduction—and encountered in examples throughout the other sections—one possible strategy for tissue barrier formation is inside, or along the surfaces, of bulk hydrogels. Their biomechanical and biochemical properties can play a major role in determining biological function, a topic on which we refer to some excellent reviews regarding hydrogel choice and design [402, 403, 404]. Here, we just briefly note that the OoCs we have reviewed throughout continue to suffer from hydrogels that are overly simplistic (e.g., gelatin) or non‐specific and batch‐variable (collagen I, matrigel). There thus remains great potential to integrate more well‐defined, tissue‐specific hydrogel materials using either mixtures of human‐derived recombinant proteins, synthetically engineered polymers, or hybrid approaches. For the alternative and commonly encountered vertical/membrane chips, the nature of the employed membranes has implications on how well they can be used to mimic the in vivo situation, in terms of material stiffness, ECM remodeling, and thickness [405]. The typically used membranes throughout our review—based on thermoplastics (affordable commercial availability) or PDMS—are thick (≳ 10 μm) and not very permeable ($\tilde{1}\%$), limiting both biochemical diffusion and direct cell–cell contact across. Although these specific limitations can be overcome with more advanced, but relatively resource‐intensive, cleanroom‐based membrane fabrication approaches, an alternative exists in the use of biomaterials to more closely approximate basement membrane [406]. Zamprogno et al., for instance, formed arrays of self‐suspended collagen/elastin membranes with a thickness of 4.5 μm (Figure 5a) [407]. A gold mesh provided structural stability and kept their hydrogel solution in place through surface tension during gelation. The researchers demonstrated the membrane's use specifically as a primary cell‐based lung model, with their hexagonal array of stretchable membranes reminiscent of alveoli. By contrast, Mou et al. pursued larger‐scale freestanding membranes by electrospinning [408]. They specifically relied on silk fibroin to confer superior mechanical properties (at the cost of remodeling ability), with a subsequent laminin coating providing human biochemical relevance. There remains, however, room for improvement, with thicknesses still significantly greater than the basement membrane's 0.1–1 μm, and a lack of compositional complexity compared to the basement membrane's tissue‐specific, layered mix of biopolymers [409].

#### 3.12.2 Measuring Barrier Integrity

One, if not the most, common readout from barrier models is barrier integrity, including electrically by TEER. It can be adapted to OoC most simply by insertion of electrode wires into chip channels; however, this makes long‐term, incubator‐stable measurement setups difficult to establish, with researchers often resorting to taking their OoC out of the incubator for measurements. Jeong et al. were the first to demonstrate minute‐scale temporal TEER resolution on a murine BBB‐OoC, albeit only over a 12 h period [413]. Some of us later demonstrated multi‐day OoC monitoring at similar temporal resolution with a human iPSC‐BBB [414]. We relied not on TEER but on the closely related electric cell‐substrate impedance sensing (ECIS). Using co‐planar interdigitated electrodes integrated directly on the cell culture membrane, this approach helped simplify the requisite electrical connections. Some efforts toward also spatial mapping are under way, employing, for example, movable electrodes or electrode arrays [415, 416]. Wong and Simmons, meanwhile, pioneered a distinct approach reliant not on ions naturally present in the media, but on small‐molecule electrochemical probes added to the media and detected on a working electrode [417]. This is a close analogue of widely used fluorescent tracer permeability assays, and could similarly be extended also to larger‐molecular‐weight probes, providing broader information on barrier leakage than (ion‐only) TEER. One clear downside of the electrode integration necessary for all of the above, however, is that it hinders microscopic inspection and imaging. A number of approaches are being investigated to overcome this, including the use of transparent indium tin oxide (ITO) [418, 419] or more recently also of poly(3,4‐ethylenedioxythiophene): polystyrene sulfonate (PEDOT:PSS; a semitransparent conductive polymer that does not require cleanroom processing) [420]. Extension of reproducible TEER to 3D vessels and especially networks also remains a largely unresolved challenge.

#### 3.12.3 Biochemical Sensors

Besides barrier integrity, OoCs can provide a unique platform for integrated real‐time monitoring of biochemical secretion, uptake, metabolism, and transport [421]. Electrochemical sensors offer a wide range of opportunities, given the many well‐established analyte detection schemes and the electrical (i.e., miniaturizable) chip interface and readout. Dornhof et al. presented an OoC platform with concurrent and continuous monitoring capabilities for oxygen, glucose, and lactate (Figure 5b) [410]. Although they demonstrated their system with breast cancer spheroids inside hydrogels, an extension with tissue barriers alongside the hydrogel surfaces can easily be envisioned. In contrast to Dornhof et al. placing their electrodes in the adjacent microfluidic channels, Utagawa et al. showcased using a gold‐coated membrane as a sensor in immediate contact with barrier tissues on chip—including nitric oxide release from HUVECs, and alkaline phosphatase activity with Caco‐2 [422, 423]. They have yet to demonstrate continuous (rather than intermittent) monitoring, however, and the lack of bio‐recognition elements presents limitations with regard to specificity. Electrochemical sensors also have certain disadvantages, including a risk of reaction products from the working and/or counter electrode affecting biological function. Optical transducers can avoid this (unless enzyme‐based), and their simple “connections” can take advantage of the inherent transparency of OoC materials (as opposed to additional electrical wires/connections). In particular, phosphorescence detection of certain chemical indicators—spot‐cast inside the OoC—has become popular for requiring only a compact instrument and an optical fiber. Izadifar et al. recently combined TEER with such optical sensor spots for media oxygenation and pH, placed on both sides of a barrier to allow for multimodal continuous monitoring [424]. Fuchs et al., meanwhile, succeeded in extending the range of phosphorescence sensing analytes [425]. They encapsulated glucose oxidase—whose reaction relies on equimolar glucose and oxygen—in a hydrogel on top of the well‐established oxygen‐sensitive optical indicator spot, and showed that thus‐measured oxygen depletion translated accurately to glucose concentration. Other optical detection methods can allow for an equally broad range of analytes as electrochemical sensors, but at the cost of higher integration complexity. The possibilities of such sensors were presented by Cognetti et al. in a vertical/membrane OoC by incorporating an array of optical ring resonators, functionalized with antibodies for multiple cytokines of interest [426]. Capture of epithelial‐secreted cytokines results in a shift in resonant wavelength of the sensor, revealing inflammatory response with minute‐by‐minute resolution.

#### 3.12.4 Media and Tissue Oxygenation

Oxygenation is often overlooked despite the large regional and developmental variations in vivo [427]. OoCs fundamentally allow for two strategies. First, oxygenation can be regulated as in traditional in vitro models by using a hypoxia incubator when employing a sufficiently gas‐permeable microfluidic construction material. This is a distinctive advantage of the widely used PDMS; given its well‐known limitations (bubble formation, small‐molecule absorption), there remains an unmet need for equally gas‐permeable materials. The second strategy is relying on perfusion of media, equilibrated to the desired oxygenation, into a gas‐impermeable OoC (e.g., most thermoplastics). This more closely resembles the in vivo circulatory system function, and more easily allows for establishing physiological cross‐barrier oxygenation gradients, the GI tract providing the most pronounced example [428]. What should not be neglected, however, is the sensitive cell seeding/attachment phase, which precludes perfusion and often results in undesired hypoxia. Without red blood cells and their hemoglobin, there is also a fundamental limit to how much oxygen can be provided through media perfusion [429]. On top of these two basic approaches, additional and more elaborate options have emerged to more accurately control oxygenation levels. Jiang et al. combined the aforementioned oxygen sensors in their thermoplastic OoCs with a flow circuit that also included dedicated PDMS‐based chips—to either let their media absorb oxygen from ambient air, or to have a liquid‐dissolved chemical scavenger absorb excess oxygen *from* their media [430]. In combination, this allowed for true closed‐loop control. Alternatively, the oxygen scavenging ability of off‐stoichiometric thiol‐ene‐epoxy, a versatile OoC fabrication material, has been demonstrated [431, 432]. With its scavenging capacity tunable based on design and processing parameters, this offers a less controlled but single‐chip opportunity for control. Santiago et al. relied instead on including a gas perfusion system in their (macro‐scale) culture chamber [433]. By applying ambient air and nitrogen on opposing sides, the tissue in between could be subjected to an oxygen gradient for modeling ischemic stroke.

#### 3.12.5 Mechanical Stimulation and Sensing

Across barrier models, controlled mechanical stimuli are a key advantage of OoCs. However, typical uniaxial stretch does not necessarily reflect the more complex in vivo mechanical cues of a given tissue that can affect factors like cell alignment, stem cell fate, and ECM remodeling [434]. Gizzi et al. extended the concept to biaxial stretch with a square culture membrane surrounded by pneumatic‐actuation chambers on all four sides [435]. More recently, radial stretch—a close match for alveoli—and even template‐based (thus theoretically arbitrary) out‐of‐plane deformation have been demonstrated, though construction of the requisite OoC and/or the external components becomes much more complex [436, 437, 438]. One common shortfall of all the above remains the use of PDMS, not always an optimal material choice. Thermoplastic elastomer materials should prove a welcome alternative here. For hydrogel‐hosted tubes or networks, Dessalles et al. also demonstrated the direct application of fluid pressure to generate radial stretch, and presented different strategies to decouple pressure and flow [439]. In parallel with stimulation, it should also be insightful to monitor the exertion or relaxation of cellular forces, which until recently remained confined to heart or muscle‐OoC. She et al. pioneered the incorporation of an MXene‐based sensing layer into a hydrogel‐based film that could function as a stretchable OoC culture support [440]. By monitoring MXene electrical resistance, they were able to track changes in lung epithelial contractility upon LPS inflammation and its drug treatment.

#### 3.12.6 3D Printing

Additive manufacturing, or 3D printing, is a powerful tool that has found broad application in enhancing or supplementing existing OoC construction techniques such as sacrificial molding, injection molding, or drop casting with a wider range of geometries [441]. A unique promise is the direct “single‐step” creation of complete 3D microfluidic architectures in which cells can be seeded. Cao et al., for instance, introduced a specialized triple‐concentric extrusion nozzle to print hollow hydrogel tubes [442]. The researchers were able to adjust wall thicknesses and permeability, and—after seeding respective primary ECs—to thereby model vascular and one‐end‐blind lymphatic capillaries. Xu et al. recently opened up an even broader range of geometries by using two‐photon‐polymerization printing of PDMS (Figure 5c) [411]. They succeeded in creating porous and flexible microvessel structures with near‐arbitrary tortuosity and entanglement, though their initial biological application remained confined to an epithelial breast cancer cell line. Another valuable—and already more widely explored—approach is bioprinting, where OoC tissue structures are printed directly using cell‐laden hydrogels [443, 444]. The benefits of bioprinting include the ability to create heterogeneous distributions of different cell types within the same space and with a high degree of control over cell ratios and amounts. A common limitation with such bioprinted systems is the continued need for a surrounding mechanically and fluidically stable enclosure created by other means. To address this, Lee et al. demonstrated an integrated process for printing both a plastic microfluidic channel with a microporous cell culture support, as well as hepatocytes in a decellularized liver ECM bioink and a HUVEC‐containing gelatin bioink [445]. Machour et al. more recently also realized a wide range of biomaterial stiffnesses by combining a regular soft, cell‐laden fibrinogen/collagen/hyaluronan bioink with a mechanically strong microparticulate ink based on synthetic polymers and hydroxyapatite to print structurally complex vascularized bone [446]. Across publications, the choices of the respective hydrogel materials again demonstrate the range and potential shortcomings of biomaterials as touched upon earlier.

#### 3.12.7 OoC Interconnects

OoCs in many if not most cases require some form of fluidic connection, be it to external pumps, downstream sensors, or other OoCs. Single‐chip system integration is of course possible, but is often complex, costly, and not easily adaptable to different scenarios. The use of tubing interconnects facilitates a high degree of flexibility and adaptability, but is also not without issues. One of these is large fluidic dead volumes that introduce time delays and possibly chemical absorption or degradation. Loskill et al. addressed this constructing specialized glass/PDMS connectors of as low as 0.05 μl dead volume that could freely and flexibly interconnect between an array of (here, simple, single‐chamber) OoC “units.” Fluid displacement (which may cause undue shear), and the practical difficulty of handling small connectors in tight spaces, remained [447]. Ong et al. attempted to alleviate such limitations in their system of modular OoC culturing chambers [448]. Instead of out‐of‐plane access ports, they constructed horizontal/in‐line ports equipped with magnets for easy snap‐on alignment and connection. Yet dead volumes are again increased (alongside construction complexity), and bubble formation at (de)connection remains a concern across all extant approaches. In both examples, moreover, OoC and interconnects were purpose‐designed to work together and would not necessarily work with other extant OoCs. There has thus been some effort to formally standardize aspects of OoC design and (inter)connectivity, also in order to facilitate an easier adoption of complex OoCs into industry, to be discussed as part of the following section.

### 3.13 Standardization and Translation

#### 3.13.1 Regulatory Perspective

Variations in chip materials, fabrication approaches, cell sources, and functional readouts introduce substantial heterogeneity between OoC systems, complicating data comparability, and undermining analytical robustness required for regulatory evaluation [449, 450]. Although the United States Food and Drug Administration has not yet formally qualified any barrier‐on‐chip system, it does recognize OoCs as promising alternatives for animal models in drug development and toxicity screenings [451]. The European Medicines Agency also puts a strong emphasis on the 3R principle (reduction, replacement, and refinement of animal studies), with OoC considered a key enabling technology. Both agencies will consider data from OoCs to support applications for new drugs and compounds, provided the applicant supplies sufficient and convincing evidence of their relevance—which can be burdensome and holds uncertainty for the applicant. A critical regulatory requirement toward broader acceptance is the precise definition of an OoC context of use—such as predicting drug‐induced liver injury [452, 453]. It is within this context of use that the first OoC Qualification Plan—for a commercial vertical/membrane liver‐OoC (akin to the one by Nawroth et al. reviewed in Section 3.7.3 [245], with the addition of stellate cells)—has recently been accepted within the FDA's Innovative Science and Technology Approaches for New Drugs Pilot Program [454]. This OoC is thus starting the final stage of the evaluation process, after which it will be considered a qualified (i.e., trusted and reliable) tool for drug development within its defined liver toxicity context of use; a handful of other OoCs are at earlier steps in the process [455].

More broadly, the integration of human‐derived (primary or iPSC) cells into OoCs must comply with general guidelines for the handling of human tissue, including informed consent and ethical committee approval as formalized in the Declaration of Helsinki [456]. Donors afflicted by impaired decision‐making are a particularly vulnerable population, as decisions rest with their legal guardians. Donor consent needs to consider not only the immediate tissue removal but also storage and sharing as well as data protection and privacy risks associated with biological materials (including re‐identification risk from multi‐omics and machine learning) [457]. For iPSCs, the scope of use deserves particular attention due to potentially sensitive applications enabled by pluripotency (e.g., neural organoids, genome editing, in vitro gametogenesis). Such considerations are part of the ISSCR Guidelines for Stem Cell Research and Clinical Translation, which thoroughly cover related best practices and quality controls from the cell culture hood to the clinic [458]. For primary materials, though many similar considerations apply, no similar/definitive guidance exists, except general Good Laboratory/Manufacturing/Clinical Practices—including controlled documentation, traceable sourcing of all materials, sterility assurance, and standardized, reproducible workflows.

#### 3.13.2 Biological Challenges of iPSCs

iPSC‐derived cells continue to face fundamental limitations (maturity, epigenetic memory; Section 2), and high costs alongside long production times constrain their translation and scalability [459]. In an effort to address these challenges, the methodologies used for differentiating iPSCs have evolved over the years (as have those for reprogramming). Over time, the field has shifted from protein‐based guidance toward small molecule‐based strategies, which offer improved stability and scalability at reduced variability and cost [460]. Still, responses to small molecules can vary between iPSC lines, even from the same donor [461]. More recently, transcription factor‐based programming and direct lineage conversion have emerged as promising next‐generation approaches. By overexpressing lineage‐defining transcription factors, these methods can bypass long differentiation protocols, produce more uniform cell populations, and provide more precise control over cell identity [461, 462]—at the expense of somewhat higher cost and accessibility. Regarding iPSC‐derived cell maturity, a wide range of approaches have been proposed and studied, including long‐term culture, reactive oxygen stressors, ionizing radiation, telomerase inhibition, genetic manipulation (e.g., progerin overexpression or telomerase reverse transcriptase knockout), or even small‐molecular maturation cocktails (targeting chromatin remodeling and calcium‐dependent transcription) [463, 464, 465, 466, 467]. These approaches elicit hallmarks of cellular aging, including DNA damage, mitochondrial dysfunction, senescence, and altered lineage‐specific markers, but their efficacy can vary by cell type. For general (rather than individualized) disease modeling, sex and ethnicity representation in iPSC availability also pose continued challenges [468]. Finally, the importance of the microenvironment that we emphasize in the context of OoC models also applies to iPSC differentiation. Whereas advanced microfluidic systems are generally resource‐prohibitive here, a simple but prime example lies in the endocrine environment, with most differentiation protocols using hormonally neutral media. Yet, for example, estrogen improves calcium handling and ion channel expression in female iPSC‐derived cardiomyocytes, while testosterone enhances the differentiation efficiency of male‐derived pancreatic beta cells [469, 470]. Continued efforts along these lines are needed to fully unlock the translational potential of iPSC technology in the context of OoC platforms.

#### 3.13.3 Technological Standards

Initial engineering standardization efforts were driven not just by OoCs but also other application areas like point‐of‐care testing that rely on not‐entirely‐dissimilar chip systems, and culminated in the formal ISO 22916:2022 for microfluidic devices (as well as an ISO 10991:2023 vocabulary) [471, 472]. It standardizes a number of preferred platform dimensions that align with established laboratory workflows (e.g., well plates, microscopy slides) as well as the crucial placement and spacing of fluidic ports. This has enabled the creation of versatile “(micro)fluidic circuit boards” to which a wide range of standard‐conforming modules can be connected as, for example, by Dekker et al. with a pressure sensor, reservoirs, valves, and so forth [473]. Vollertsen et al. proceeded to demonstrate this type of platform for highly parallelized cell culture with 64 individually controlled OoC chambers (Figure 5d) [412]. ISO standardization is ultimately an important step in achieving OoC modularity and interoperability across academic labs and commercial manufacturers, allowing researchers to more easily realize new OoC functionality based on existing OoC building blocks. In turn, this also promises to facilitate better comparison and reproducibility across OoC studies. Work also continues to standardize additional and specific aspects of OoCs (from terminology to biology, from engineering to experimental analysis), with a dedicated ISO working group for Microphysiological systems and Organ‐on‐Chip established in 2024 [474].

#### 3.13.4 Commercial OoC Platforms

As OoCs have become a more popular avenue of research, commercialization of OoC platforms and tools has likewise increased, and we will mention some noteworthy examples below. Such commercial platforms are attractive for primarily three reasons: First, they eliminate the need, and therefore the required equipment and expertise, to produce microfluidic devices in‐house. Second, even if not following existing ISO standards (though some do), commercial OoCs provide a more “standardized” platform that benefits reproducibility both within a lab over time, and between different labs. Third, some companies' semi‐automated liquid handling systems further increase reproducibility and throughput while reducing required microfluidic operation expertise. This makes them very attractive to biomedical researchers and to the pharmaceutical industry. The trade‐offs—especially for vertical/membrane or also sensing/stimulation‐integrated OoCs—are high cost and an intrinsically limited design space. It is critical to evaluate whether the microenvironment provided by a commercial OoC platform can deliver the specific microenvironment needed for the tissue and biological question of interest.

The “classical” PDMS‐based vertical/membrane chip design first termed OoC, developed by Huh et al. (Section 3.6.2 [186]; see also Figure 2c,d) has been commercialized by Emulate (Chip‐S1). This is the commercial OoC we most commonly encountered throughout our review (though far short of a majority). Emulate provides a custom system for active perfusion, and connection to other pump systems is relatively simple, but neither the chip nor its connectors are ISO‐compliant. Vertical/membrane OoCs with ISO‐compliant footprint and/or interconnects (albeit non‐stretchable) are available on the market from companies like Dynamic42 (BC001), BeOnChip (Be‐doubleflow), and others. Emulate has notably expanded their portfolio recently toward a higher‐density well‐plate format (Chip‐Array; integrating 12 “organs”).

Such higher‐density well‐plate formats are more common for lateral/hydrogel designs. The Mimetas OrganoPlate (Figure 3e) and AIM biotech idenTx Plate offer, respectively, 64 or 40 triple‐compartment “organs”—designed solely for gravity‐driven perfusion (controlled by a programmable rocking platform). The similar but microscopy‐slide‐format 3‐“organ” idenTx Chip is notable for also being amenable to tubing/pump connection, though adapters (sold separately) are needed for ISO‐conforming Luer. Sharing the same footprint and “organ” number, the BiomimiX uBeat conversely sacrifices pump connection for flexible materials and a pressure channel running underneath the tissues, applying up to 10% mechanical deformation. OoC systems offering integrated pumps are also available, most commonly as multi‐OoCs. TissUse HUMIMIC chips consist of 2, 3, or 4 microfluidically‐interconnected, Transwell‐like culture chambers (Figure 4b). The approach is similar to CNBio's PhysioMimix, which are designed to host 6 barrier–liver co‐cultures in a well‐plate format (a version with 12 individual barrier chambers is also available). This is only highlighting a fraction of commercial OoCs, and with a focus on “standard” designs. Given increasing interest in barrier–organoid co‐culture models, corresponding options have been released recently, including by some of the above companies. Other companies specialize, such as Alveolix toward lung models, with their AX12 incorporating 12 air‐liquid interface “organs” with biomechanical stimulation ability. Finally, a number of commercial OoCs are also kept in‐house and offered as a service only, Hesperos (based on the very first microfluidic cell culture system [187]) being a notable example.

## 4 Conclusion

Impressive advances in OoC and iPSC technology in recent years have enabled the development of human barrier models with superior predictive value for disease modeling and drug testing. By providing dynamic and physiologically relevant microenvironments that are unattainable in conventional static models, OoC platforms have proven their utility across a wide range of biological systems and disease contexts. In parallel, progress in iPSC differentiation protocols has made it possible to generate crucial cell types, including those difficult to obtain primary material for (e.g., BBB). Now, the integration of patient‐derived and iPSC‐based cells with tailored tissue interfaces, mechanical cues, and flow regimes in OoCs has helped to address limitations of both iPSC‐derived cells (immaturity) and primary cells (dedifferentiation). This has led to great momentum in personalization of disease modeling and therapeutic screening, with notable examples ranging from dermal‐OoCs incorporating immune and stromal co‐cultures to model autoimmune and chronic inflammatory skin diseases, to airway models incorporating cyclic stretch and airflow for investigating conditions such as cystic fibrosis and allergic diseases, to multicellular genotypic models of vascular Progeria or Huntington's.

However, progress remains uneven, with disparities illustrating both the promise and the current bottlenecks in building physiologically complete and personalized models (cf. Table 1). For several tissues, including parts of the reproductive tract, lymphoid organs, or renal and aural systems—where some of the relevant primary cells are not necessarily easily or commonly available—models incorporating iPSC‐derived cells remain in early stages or have yet to be established. This is not to mention the limited progress on reproducing regional heterogeneity with iPSC‐derived cells. Yet there is exceptional potential even in combining existing iPSC‐based models or primary cells—a practical and valuable strategy for more accessible tissues such as skin or oral mucosa—with existing OoC technology. As comparing between the different sections of our review shows, most of the fundamental barrier architectures and features are shared across multiple tissues and can be recapitulated with similar if not identical OoCs (though the examples depicted in Figures 2 and 3 were explicitly chosen to showcase a wide range of approaches, this still becomes apparent even from their limited selection). The experimental handling does continue to present additional practical challenges compared to well‐plate culture. However, a broad range of commercial chips (including a few at price points not too different from Transwells) is now available, and a sizable academic OoC engineering community (some of them more likely than not in your proximity) has by now established itself. Standardization and reproducibility along with regulatory acceptance are also advancing, albeit slowly. There are thus few barriers to realizing the untapped potential for genotypic OoC models—already ranging, as we've encountered, from personalized therapeutic response assessment to discordant models to unravel mechanisms of disease—for biomedical researchers with the right biological material.

At the same time, there is room for continued engineering innovations. We have highlighted recent advances in monolithic 3D printing or oxygen management that are still in their infancy with regards to OoCs in general, and technologies for real‐time biochemical monitoring or thin and flexible basement membrane mimics that have yet to find their way into personalizable OoC disease models, to enhance physiological relevance and data richness. With multi‐organ systems, helped along by efforts in modularization and standardization, we also see OoCs continuing to advance toward capturing systemic effects that have otherwise remained the domain of animal models (in spite of their non‐human shortcomings). Ultimately, as these multidisciplinary efforts in both biology and engineering continue to converge, the next generation of barrier models is poised to make physiologically relevant, genotypic disease modeling increasingly accessible—bringing us closer to a future of personalized medicine.

## Conflicts of Interest

The authors have no potential conflict of interests to declare.

## Data Availability

The authors have nothing to report.
